# Combining laboratory and mathematical models to infer mechanisms underlying kinetic changes in macrophage susceptibility to an RNA virus

**DOI:** 10.1186/s12918-016-0345-5

**Published:** 2016-10-22

**Authors:** Andrea Doeschl-Wilson, Alison Wilson, Jens Nielsen, Hans Nauwynck, Alan Archibald, Tahar Ait-Ali

**Affiliations:** 1 The Roslin Institute & R(D)SVS, University of Edinburgh, Easter Bush, Edinburgh, UK; 2 Department of Mircrobiological Diagnostics and Virology, Statens Serum Institute, Copenhagen, Denmark; 3 Department of Virology, Parasitology and Immunity, Ghent University, Ghent, Belgium

**Keywords:** PRRSV, CD163, Alveolar macrophages, Host cell susceptibility, Mathematical model, Infection dynamics, Statistical inference, Host-pathogen interaction, Pigs

## Abstract

**Background:**

Macrophages are essential to innate immunity against many pathogens, but some pathogens also target macrophages as routes to infection. The Porcine Reproductive and Respiratory Syndrome virus (PRRSV) is an RNA virus that infects porcine alveolar macrophages (PAMs) causing devastating impact on global pig production. Identifying the cellular mechanisms that mediate PAM susceptibility to the virus is crucial for developing effective interventions. Previous evidence suggests that the scavenger receptor CD163 is essential for productive infection of PAMs with PRRSV. Here we use an integrative in-vitro–*in-silico* modelling approach to determine whether and how PAM susceptibility to PRRSV changes over time, to assess the role of CD163 expression on such changes, and to infer other potential causative mechanisms altering cell susceptibility.

**Results:**

Our in-vitro experiment showed that PAM susceptibility to PRRSV changed considerably over incubation time. Moreover, an increasing proportion of PAMs apparently lacking CD163 were found susceptible to PRRSV at the later incubation stages, thus conflicting with current understanding that CD163 is essential for productive infection of PAMs with PRRSV. We developed process based dynamic mathematical models and fitted these to the data to assess alternative hypotheses regarding potential underlying mechanisms for the observed susceptibility and biomarker trends. The models informed by our data support the hypothesis that although CD163 may have enhanced cell susceptibility, it was not essential for productive infection in our study. Instead the models promote the existence of a reversible cellular state, such as macrophage polarization, mediated in a density dependent manner by autocrine factors, to be responsible for the observed kinetics in cell susceptibility.

**Conclusions:**

Our dynamic model–inference approach provides strong support that PAM susceptibility to the PRRS virus is transient, reversible and can be mediated by compounds produced by the target cells themselves, and that these can render PAMs lacking the CD163 receptor susceptible to PRRSV. The results have implications for the development of therapeutics aiming to boost target cell resistance and prompt future investigation of dynamic changes in macrophage susceptibility to PRRSV and other viruses.

**Electronic supplementary material:**

The online version of this article (doi:10.1186/s12918-016-0345-5) contains supplementary material, which is available to authorized users.

## Background

Alveolar macrophages are primary effectors of innate immunity against respiratory pathogens [1]. Some pathogens however target alveolar macrophages to initiate infections that produce severe disease in humans and livestock [2–4]. Alveolar macrophages are not homogeneous, and may vary in their response to pathogens [5, 6]. Identifying the cellular mechanisms that alter the susceptibility of these cells to the pathogen in question is crucial for developing effective interventions.

This question is particular relevant for Porcine Reproductive and Respiratory Syndrome (PRRS), one of the most devastating pig diseases worldwide caused by the PRRS virus (PRRSV) [6]. PRRSV is a 15 kb positive-strand RNA virus of the *Arterivirdidae*, order *Nidovirales* family that infects subpopulations of porcine alveolar macrophages (PAMs) [6]. The PRRSV replication cycle in PAMs is relatively short, namely between 12 and 18 h post infection [7, 8]. Regardless of the strain genotype and pathogenicity, the virus produces a rapid increase towards peak virus load at around 5–10 days post infection, followed by a more gradual decline until clearance at 3 to 10 weeks post infection [9–11]. Previous studies have demonstrated substantial inter-host variation in the rate of post-peak decline [12]. Understanding the underlying factors controlling the rate of virus load decline after peak levels have been reached would be highly desirable for the development of intervention strategies. However, to date it is still a mystery what causes the virus load decline in the first place.

In the absence of the typical contenders for reducing virus load within a host, it has been postulated that change in the permissiveness of resident PAMs to PRRSV over the time course of infection may be responsible for the observed post-peak virus load decline [13, 14]. Indeed, previous in-vitro studies have shown that susceptibility of freshly isolated PAMs increases within 4 days of culture [8, 15]. However, it is not known whether the susceptibility of PAMs can also decline, and what may modulate this trend.

Three cell molecules have been shown to play an important role in the productive PRRSV infection of macrophages: Heparan sulphate, involved in PRRSV binding (e.g. [16, 17]), Sialoadhesin (CD169), involved in virus binding and internalisation [18], and the scavenger receptor CD163, found to be essential for viral uncoating [19]. Neither Heparan sulphate nor Sialoadhesin [20] are essential for productive PRRSV infection of macrophages, but gain-of-function experiments have found CD163 to be both necessary and sufficient to render a variety of non-susceptible cell lines competent for PRRSV infection [21, 22]. Hence, altered CD163 expression with time could contribute to changes in PRRSV susceptibility at the single cell level.

Measuring changes in susceptibility of alveolar macrophages to pathogens over the time course of infection in-vivo is challenging. In-vitro experiments, in contrast, allow close inspection of cellular properties in controlled environmental settings. When coupled with in-silico models via statistical inference, novel insights into dynamic properties and underlying mechanisms of key infection characteristics that are difficult to measure empirically (such as change in host cell susceptibility) can be obtained (e.g. [23, 24]). In this study we combine in-vitro and in-silico infection models to determine whether and how the susceptibility of cultured PAMs to PRRSV changes over time and to examine possible functional modulation of CD163. In particular, we develop process based mathematical models to evaluate alternative hypotheses about the role of CD163 in PRRSV infection dynamics that emerge from our in-vitro experiment, and by fitting the models to the experimental data, infer the nature of potential cellular processes underlying the observed susceptibility trends of PAMs.

**Fig. 1:**
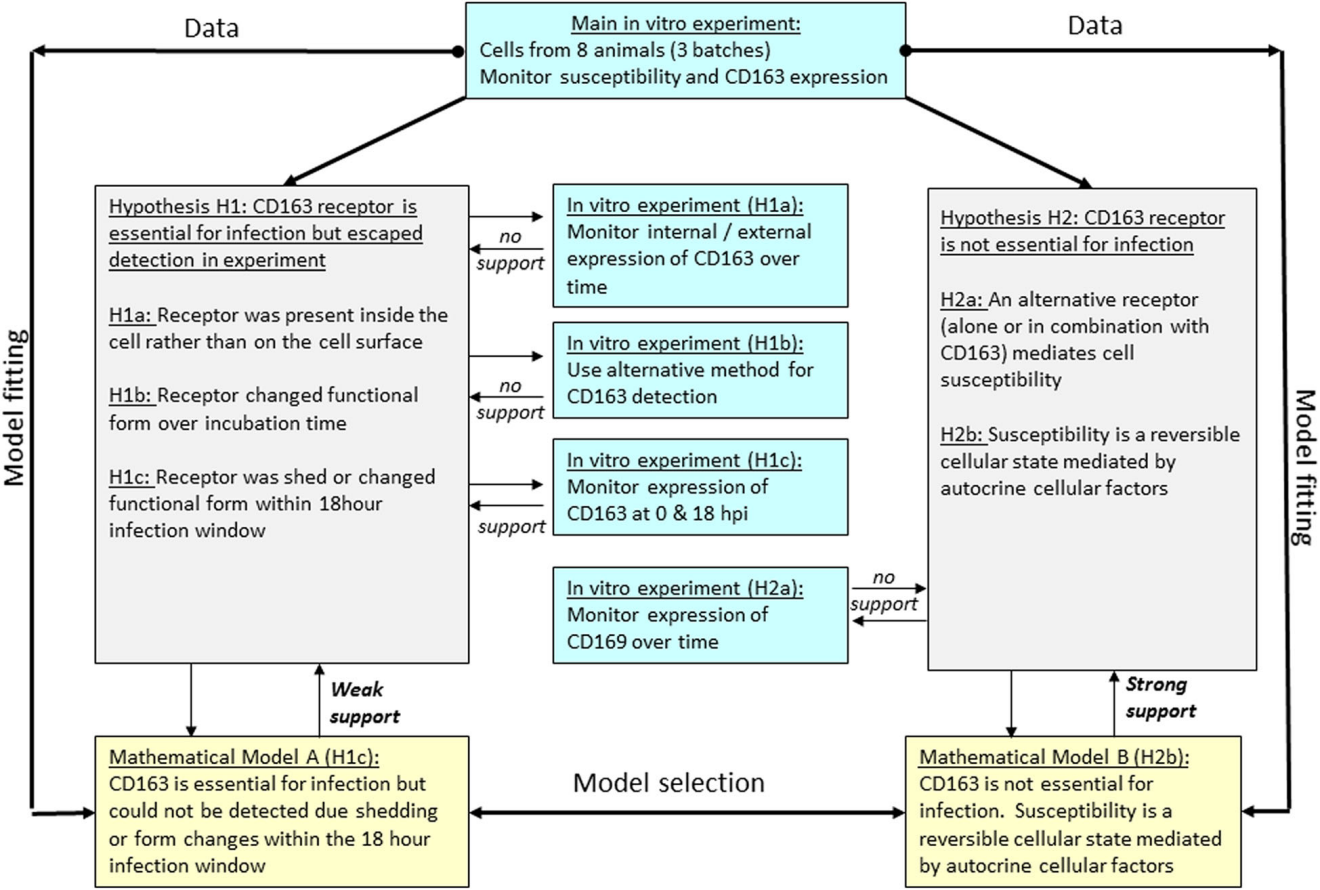
Schematic diagram of the work-, information- and decision flow adopted in this study

The iterative workflow adopted in this study is outlined in the schematic diagram of Fig. 1. The paper is organised accordingly as follows. In the Methods section we describe the in-vitro experiment and the statistical data analysis, as well as the fitting and selection process adopted for the process based mathematical models. The Results section starts with a description of the key experimental findings. Based on these, two alternative hypotheses for the underlying cellular processes mediating PAM susceptibility over time emerged (denoted as Hypothesis H1 and H2 in Fig. 1), which were further explored in subsequent in-vitro experiments (referred to as H1a-H1c and H2a in Fig. 1). The combined results then led to the development of two distinct mathematical models (denoted as mathematical model A & B in Fig. 1) representing alternative mechanisms underlying the observed changes in cell susceptibility and bio-marker dynamics. Finally, we fit the models to the data (i.e. link the top and bottom boxes in Fig. 1) to infer the nature of the potential dynamic processes regulating PAM susceptibility to the PRRS virus over time. In the Discussion section we compare our findings with existing evidence from other studies of the regulation of PAM susceptibility to PRRSV and other viruses. We conclude by pointing out the implications of our findings for future research in within host infection dynamics.

## Methods

### In vitro experiment and statistical analysis

We monitored the trajectories of virus infection together with CD163 marker dynamics of PAMs extracted from eight healthy pigs from three separate experimental batches with different genetic backgrounds,to sample diverse responses. Batches 1 and 3 comprised 3 pigs and batch 2 comprised two pigs. The PAMs were extracted via broncho-alveolar lung lavage, as described in Additional file 1. Cells harvested from each pig were assigned to one of two different culture replicates with one replicate subjected to infection with a European type I PRRSV strain (UK H2 PRRSV; multiplicity of infection above 3) and the other replicate subjected to mock-infection with the equivalent volume of growth medium.

To assess changes in PAM susceptibility and CD163 expression, cells in each replicate were distributed into separate cultures each comprising 5 × 10^6^ cells with corresponding incubation periods of 0, 1, 2, 4, 6, 8 and 9 days (or, more precisely, for 4, 28, 52, 100, 148, 196 and 220 h post extraction from freezer), respectively, before either PRRSV or mock infection. For batch 1, only 5 incubation days were used (omitting days 4 and 8). Measures obtained from the cell media at each incubation time indicated that pH, glucose and lactate concentration, and essential amino acids were stable within the 9 incubation days. Furthermore, cell viability in every culture was above 80 %, providing a sufficient amount of viable cells in each culture to quantify changes in PAM susceptibility and CD163 expression.

Immediately prior to (mock) infection, the growth medium was removed. One hour post infection, the virus/medium was removed and the growth medium was replaced, and each culture was left for further 18 h to allow for productive infection with one round of virus replication [8, 18, 26]. The 18 h infection period together with a multiplicity of infection above three in all cultures ensured that all susceptible cells had sufficient exposure to the virus and that infected cells could be reliably detected in the subsequent flow cytometry [26, 27]. After 18hpi, cells were washed, fixed where appropriate (BD Cell Fix, BD Biosciences) and viable cells were stained with monoclonal antibodies to assess they had been infected with PRRSV (SDOW17-FITC, Rural Technologies) and/or expression of CD163 (CD163-PE, clone 2A10/11, AbDseroTec) using flow-cytometry as described in Additional file 1.

The experiment provided for every individual pig longitudinal measures of i) the proportions of CD163 positive cells from the mock infected samples, and for the infected samples, the proportions of (ii) non-infected CD163 positive cells, (iii) non-infected CD163 negative cells, (iv) infected CD163 positive cells and (v) infected CD163 negative cells, obtained at seven (five for pigs from batch 1) sampling times T_i_ + 18 h, where T_i_ = i × 24 + 4 h, i = {0, 1, 2, 4, 6, 8, 9}, represent the different cell incubation times prior to (mock) infection. Statistical analyses of observed and predicted trends in PAM susceptibility and in the cell surface expression of CD163 were performed using linear mixed models implemented with the SAS *proc mixed* (SAS 9.3) with proportions of infected cells and/or cells classified as CD163 positive (negative) as response variables. The statistical models included batch, infection type (mock or PRRSV infection) and incubation day, and all significant interactions as fixed effects, and pig within batch as random effect. Normality checks on the model residuals were carried out to ensure the validity of using proportions as response variables.

### Mathematical models

#### Modelling approach

The experiment produced alternative hypotheses with regards to the role of CD163 in the infection dynamics and for the underlying dynamic processes (see Fig. 1 and Results section). To investigate these further, we developed alternative deterministic mathematical models representing diverse sets of dynamic processes and their interactions underlying the observed time trends in cell susceptibility and biomarker expression.

For the model building the principle of Ockham’s razor was applied, i.e. the aim was to develop the simplest models possible requiring the minimum set of assumptions, biological processes and variables necessary that can reproduce the experimental results with identifiable model parameters. Simultaneous to minimising the set of model processes and variables, we maximised model flexibility by allowing the rates of the biological processes to be potentially cell state dependent. This allowed us to test statistically the role of CD163 and other factors in the infection dynamics. Furthermore, to avoid bias processes that were common in alternative models (e.g. decay rates and density dependent effects) were represented by the same mathematical functions.

Dynamic processes were represented by systems of first order ordinary differential equations (ODEs) with initial conditions (IC) specified by the experimental conditions. Solutions for the model variables over time were obtained using Matlab’s numerical ode45 solver (www.mathworks.com).

#### Model fitting and identifiability analysis

A differential evolutionary algorithm with a weighted least squares fit statistics (population size 12, amplification factor F = 0.4 for mutant generation, cross-over constant 0.5, minimum number of generations 500,000) [27, 28] was used to fit the alternative mathematical models to the data. Given that the pigs in these experiments came from different genetic backgrounds, the models were fit to data (combining all samples) from each individual pig separately rather than to all pigs pooled (thus considering each pig as a random sample from different populations). This allowed us to assess whether the same model provides a consistently better fit for all eight individuals and to simultaneously gain insight into the underlying mechanisms responsible for the observed differences in time trends in susceptibility and biomarker expression between individual animals and batches. All experimental data outlined in (i) to (v) above were included in the fitting criteria. The final model parameter values were chosen based on the best weighted least squares fit of the models to the data, i.e. the differential evolutionary algorithm minimised the root mean square difference (RMS_j_) between the data and the model predictions for each individual pig j, given by

1 $$ RM{S}_j=\sqrt{\frac{1}{5{n}_j}{\displaystyle {\sum}_{k=1}^5{\displaystyle {\sum}_i^{n_j}{\left({y}_{jki}-{\widehat{y}}_{jki}\right)}^2}}} $$

Where *y*_*jki*_ refers to the i’th measurement of measure k (associated with (i) to (v) as defined above) for individual *j*, *n*_*j*_ is the number of time points T_i_ at which measurements (i) to (v) were available for individual *j*, and, and *ŷ*_*jki*_ are the corresponding model predictions.

To ensure convergence to the global (rather than local) optimum parameter combination, the differential evolutionary algorithm was run three times with different sets of starting values for each model parameter. The fitting process was terminated when convergence was achieved (i.e. parameter estimates for each individual differed by less than 5 % over 1000 subsequent iterations and by less than 10 % between different computational replicates). In the rare case (i.e. one pig) where convergence was not achieved within 1,000,000 iterations, the search parameters of the computational algorithm were modified to allow for more extensive search through the parameter space and, after using the solution after 1,000,000 iterations corresponding to the best model fit as new starting values, the algorithm was run for another 100,000 iterations.

By definition, mechanistic models representing processes that are difficult to measure are often over-parameterized resulting in poorly identifiable or non-identifiable model parameters [29, 30]. Poor identifiability manifested itself in convergence issues during model fitting and ambiguous parameter estimates or infinite confidence intervals [30]. The two strategies adopted to overcome identifiability problems consisted of (i) restricting the number of model parameters by choosing mathematical functions with the fewest parameters, and (ii) partitioning the model parameters into subsets of parameters to be estimated from the data and subsets of parameters fixed at a priori values, ensuring that the remaining parameters are identifiable. Parameter subset selection was done by calculating parameter importance based on sensitivity analysis and calculation of collinearity indices to assess near-linear dependence of parameter subsets, and by iteratively performing estimation on selected subsets until convergence was achieved [29], as outlined in detail in Additional file 2.

#### Model comparison and validation

To statistically compare the fits of alternative mathematical models, the Bayesian Information Criterion (BIC) was calculated for every model and every pig according to

2 $$ BI{C}_j={d}_j \ln \left(\frac{RS{S}_j}{d_j}\right)+m \ln \left({d}_j\right) $$

Where *RSS*_*j*_ is the sum of squared residuals (i.e. *RSS*_*j*_ = *n*_*j*_ *RMS*_*j*_^2^) for individual *j*, and *m* and *d*_*j*_ are the number of model parameters and data points (*d*_*j*_ = 5*n*_*j*_), respectively. Thus, a smaller BIC corresponds to a better model fit. Additional statistics used for model comparison included for every individual *j*, the (i) coefficient of determination *R*_*adj*,*j*_^2^, adjusted for the number of model parameters to compensate for overfitting, calculated as

3 $$ {R}_{adj,j}^2= \max \left(0,1-\frac{\left({d}_j-1\right)}{\left({d}_j-m\right)}\left(1-{R}_j^2\right)\right) $$

Where *R*_*j*_^2^ is the square of the correlation coefficient between the observed and predicted data for individual *j*, (ii) the prediction root mean square error *RMS*_*j*_ defined in (1), and the total bias *Bias*_*j*_ calculated as the sum of bias in each of the five model fit criteria outlined above:

4 $$ Bia{s}_j=\frac{1}{5{n}_j}{\displaystyle {\sum}_{k=1}^5{\displaystyle {\sum}_i^{n_j}\left({y}_{jki}-{\widehat{y}}_{jki}\right)}} $$

Furthermore, in order to test whether a simpler model with reduced number of parameters provided a statistically significant superior fit than the more complex model, a log-likelihood ratio test with threshold *p* < 0.05 was used by transforming the BIC difference between two models into the log-likelihood ratio test statistics as outlined in [12]. Note that the log-likelihood ratio test can only be applied to nested models and could thus not be used to statistically compare models representing different biological processes underlying the infection dynamics.

To provide insight into the predictive ability of each mathematical model, the parameter estimates of one predictor individual at a time, obtained by fitting the mathematical models to the data of this predictor, were used to predict the dynamic trends in cell susceptibility and CD163 expression of the seven other individuals (validation set) at the observation times T_i_. The discrepancy between the model predictions based on predictor individual l and the observations for validation individual j were then assessed using the summary statistics *RMS*, *R*_*adj*_^2^ and total *Bias* as defined above, with predictions and observations from the corresponding predictor and validation individuals, e.g.

$$ RM{S}_{lj}=\sqrt{\frac{1}{5{n}_{lj}}{\displaystyle {\sum}_{k=1}^5{\displaystyle {\sum}_i^{n_{lj}}{\left({y}_{jki}-{\widehat{y}}_{lki}\right)}^2}}} $$

where *y*_*jki*_ refers to the i’th measurement of measure k for the validation individual *j* and *ŷ*_*lki*_ refers to the corresponding model predictions for individual j obtained by parameter estimates for the predictor individual *l* and *n*_*lj*_ is the number of time points T_i_ at which measurements (i) to (v) were available for individuals *l* and *j.*

## Results

### In-vitro experiment

#### PAM susceptibility changes over time and is not mirrored by changes in the expression of CD163

**Fig. 2:**
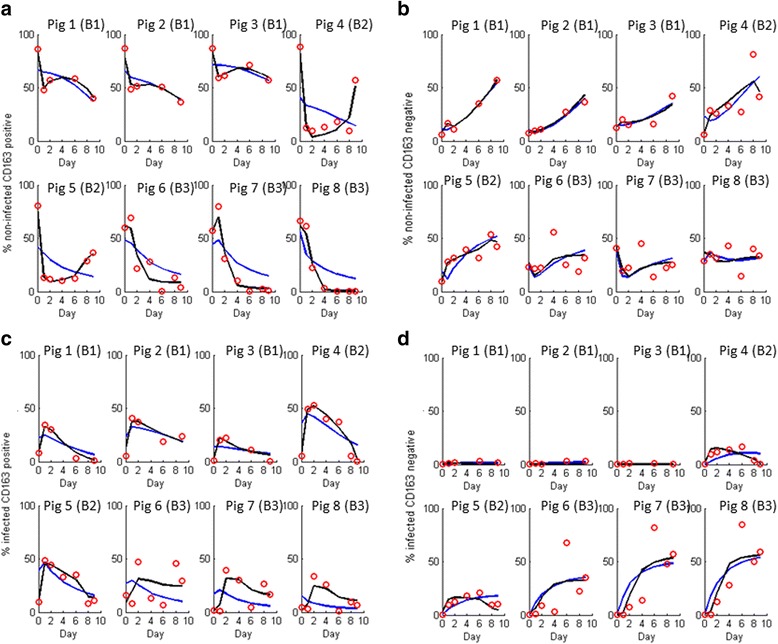
Experimental data and predictions of mathematical models A and B. Experimental data for 8 pigs from 3 batches (B1–B3) are depicted in *red circles*, and corresponding predictions from the mathematical models A and B are shown in *blue* and *black lines*, respectively. (**a **& **b**) percentage of non-infected PAMs classified as CD163 positive (**a**) and CD163 negative (**b**), respectively; (**c **& **d**) percentage of infected PAMs classified as CD163 positive (**c**) and CD163 negative (**d**), respectively. For model B, equal switching rates for CD163 positive and CD163 negative cells was assumed as this corresponded to the best statistical fit

**Fig. 3:**
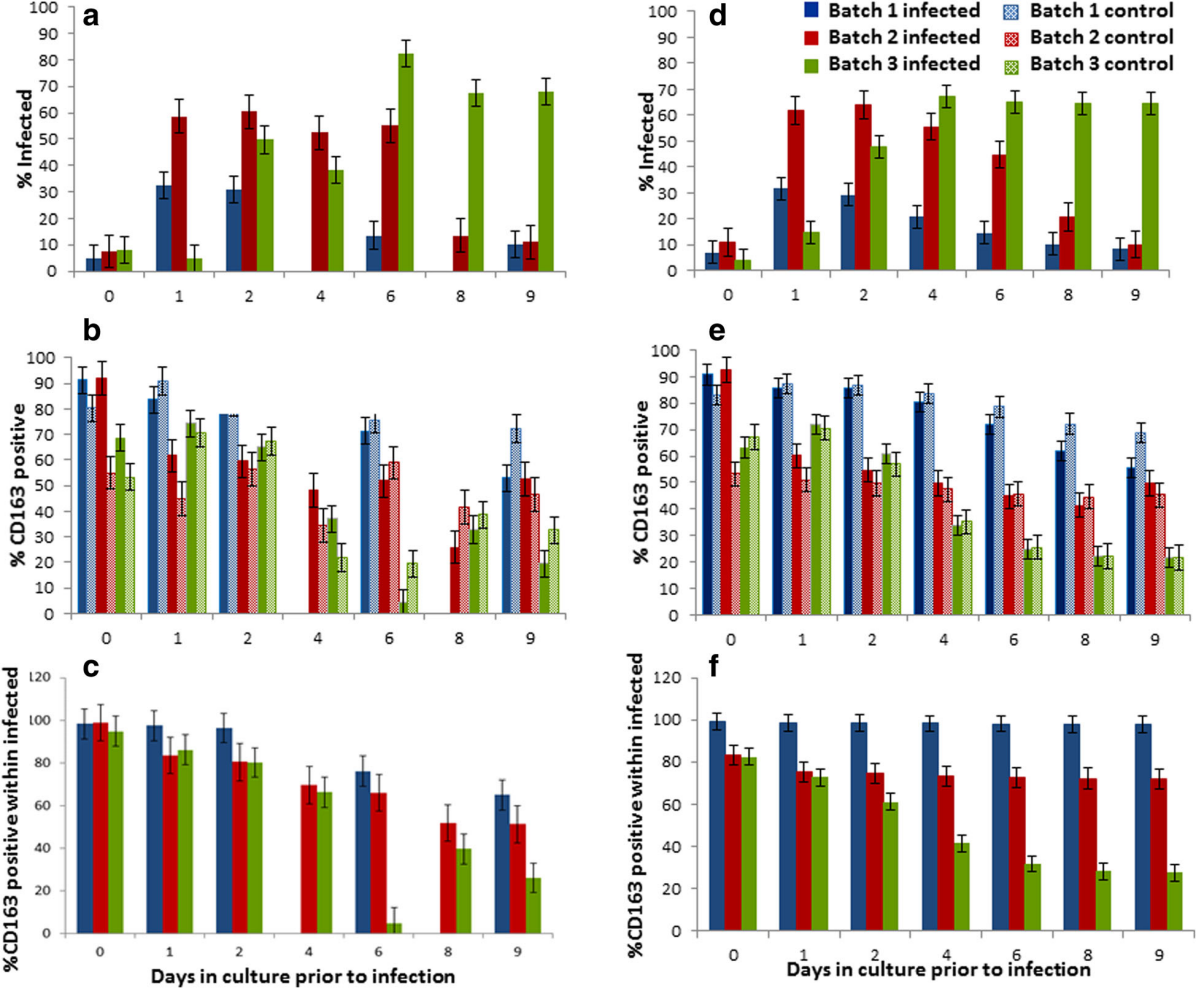
Susceptibility and CD163 dynamics. Batch specific least square mean (LSM) percentages and standard errors obtained from the experimental data (**a**–**c**) and from the predictions of model B (**d**–**f**) obtained by linear mixed model analysis. Batches 1 and 3 consisted of three biological replicates (i.e. cells from three different pigs), and batch 2 comprised two biological replicates. The estimates refer to the proportion of PAMs classified as infected (**a **& **d**), CD163 positive (**b **& **e**), and CD163 positive within the set of infected cells (**c **& **f**), after incubation for 0, 1, 2, 4, 6, 8 and 9 days, respectively, before PRRSV (infected) or mock-infection (control). Estimates refer to 18 h post (mock) infection. Predictions refer to refined model B with assumption AS1 (i.e. switching rates independent of CD163, σ_1,max_ = σ_2,max_ = σ_max_)

Susceptibility of PAMs to PRRSV changed considerably over incubation time in all samples and batches (Figs. 2 and 3a). The actual trends differed substantially between individuals (Fig. 2) and also between batches (Fig. 3), as indicated by a significant batch by incubation day interaction in the statistical linear mixed model (*p* < 0.0001). Common to all individuals and batches, PAM susceptibility was lowest at day 0, with less than 10 % of cells becoming infected. For batches 1 and 2, susceptibility had increased considerably by day 1 to least square mean peak levels around 32 % (SE = 5.1 %) and 59 % (SE = 6.3 %), respectively, where it plateaued for several days before reducing to considerably lower levels at the later incubation stages (Fig. 3a). For batch 3, in contrast, PAM susceptibility to PRRSV increased more gradually and only reached its peak level at day 6, where over 80 % of cells had become infected. In contrast to batches 1 and 2, susceptibility in batch 3 remained high at the later incubation days with least square mean values consistently above 70 % (Fig. 3a).

The percentage of PAMs expressing CD163 on the cell surface showed an overall declining trend with increasing incubation time (Fig. 3b). The rates and levels of decline differed between batches and infection groups, as shown by statistically significant batch × day and group × day interactions in the statistical model (*p* < 0.0001). Batch 3, which had the highest overall PAM susceptibility (Fig. 3a), had the lowest percentage of CD163 positive cells (Fig. 3b).

#### The relationship between host cell susceptibility and CD163 expression is complex

The observed discrepancy between the time trends in susceptibility and in the expression of CD163 (Figs. 2 and 3) indicates that changes in host cell susceptibility to PRRSV was not fully regulated by CD163, alluding thus to a complex relationship between CD163 and PAM susceptibility. In particular, at incubation day 0, the vast majority of PAMs expressed CD163 (Fig. 3b), but less than 10 % of cells had become infected (Fig. 3a). In contrast, on day 6 of batch 3, the majority of cells did not express CD163 at detectable level yet 80 % of cells had become infected. Indeed, in all infected replicates, there was an apparent increasing tropism of PRRSV towards cells that seemingly lacked the receptor as incubation time increased (Figs. 2d and 3c).

The significant infection group × day interaction in the statistical models for CD163 kinetics indicates different kinetic trends in these biomarkers between PRRSV- and mock infected samples. Indeed, at the early incubation stages, expression of CD163 tended to be higher in the PRRSV infected compared to mock infected samples, whereas at the later incubation stages, the opposite was true (Fig. 3b). These results suggest that CD163 not only influences PAM susceptibility to PRRSV, but also PRRSV infection may also influence the kinetics of CD163 expression or shedding.

### Hypotheses and mathematical models emerging from the in-vitro studies

Our observations stand in apparent conflict with evidence from static gain of function experiments that CD163 is essential for productive infection of PAMs with PRRSV [21, 22]. The following two alternative hypotheses emerged, which were further explored with subsequent in-vitro experiments and with the help of mathematical models of infection and biomarker dynamics as outlined below (see also workflow in Fig. 1).

#### Hypothesis H1 and corresponding mathematical model A: CD163 is essential for PRRSV infection, but escaped detection by the mono-clonal antibodies

There are several potential reasons why CD163 may have escaped detection by the monoclonal antibodies in our experiment. Firstly, the monoclonal antibodies (mABs) used for CD163 detection only bind to receptors on the cell surface. However, some PAMs may have expressed CD163 internally rather than on the cell surface, and may thus have been erroneously classified as CD163 negative in the flow cytometry. PRRSV co-localises with CD163 in the early endosomes [31], indicating that expression of CD163 inside the cell rather than on the cell surface is critical for permissiveness. Thus some of the PAMs classified as CD163 negative may have been indeed susceptible to PRRSV if they expressed the receptor internally (Hypothesis H1a, Fig. 1). Alternatively, the CD163 receptor may have undergone changes in its functional form over prolonged incubation periods thus escaping detection with the mABs if these changes occured in the mAB binding domain (Hypothesis H1b, Fig. 1). Such form changes may indeed explain the increase of infected cells classified as CD163 negative predominantly observed at the later incubation stages. Finally, PAMs classified as CD163 negative at the observation time 18 hpi may have expressed the receptor at the time of infection, but may have shed the receptor or the mAB binding domain within the 18 h (mock) infection period, thus also escaping detection due to the time lag between infection and observation (Hypothesis H1c). To assess these hypotheses, additional in-vitro experiments (described in Additional file 3) were carried out using additionally available PAMs from pigs in some experimental batches. The results of these small scale experiments do not support hypotheses H1a&b that CD163 was present at the time of cell screening but had escaped detection by the mABs used in the main experiment (Tables A and B in Additional file 3). However, the percentage of CD163 positive cells was found significantly lower at 18 hpi compared to 0 hpi (Table C in Additional file 3), thus indicating potential shedding or form change in the CD163 receptor within the 18 h infection period. Furthermore, the differences were more pronounced in the infected than in the mock infected samples, and at later incubation stages, indicating that both PRRSV infection and prolonged incubation may promote receptor shedding or form changes. Based on the combined experimental evidence we developed mathematical model A outlined below to further investigate hypothesis H1c, according to which CD163 is indeed essential for productive infection of PAMs with PRRSV, but had escaped detection due to shedding or form changes of the receptor over the 18 h infection period (Fig. 1).

**Fig. 4:**
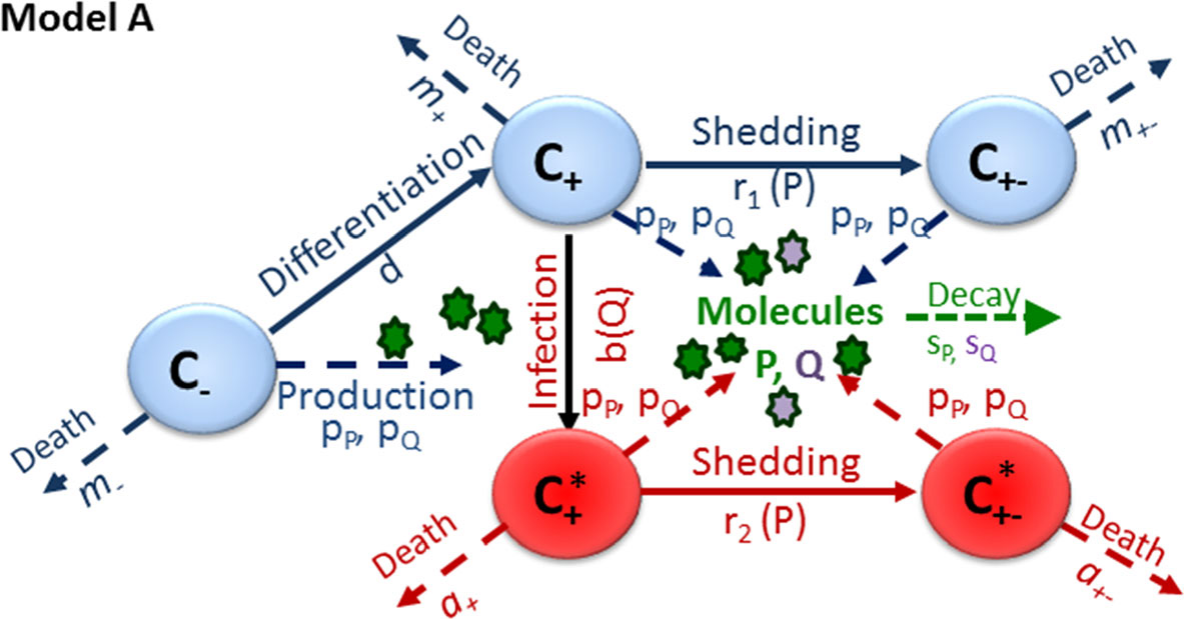
Schematic figure of model A. Components in blue, green and purple refer to the cell types and biological processes represented at all stages in the experiment (i.e. prior and post infection with PRRSV or mock agent). Components in red show additional cell types and processes after introduction of PRRSV into the cell cultures. *C*_*−*_ and *C*_*+*_ represent PAMs without/with the CD163 receptor on the cell surface, respectively, while *C*_*+−*_ represent cells that have shed the CD163 receptor. Only *C*_*+*_ cells are assumed susceptible to PRRSV. Cells indicated with ‘*’ denote infected cells. See main text for more detailed description of the model, Additional file 4 for the mathematical representation of the model, and Additional file 5 for a description of the model parameters

Model A, illustrated in Fig. 4, assumes that CD163 is essential for productive PRRSV infection, but that shedding of the receptor or the mAB binding domain within the 18 h infection period may be responsible for the high prevalence of infected CD163 negative cells that was observed 18 hpi in some samples (Figs. 2d and 3c). According to model A, PAMs can have one of three states prior to infection (or during mock infection): a priori CD163 negative, CD163 positive and CD163 negative after shedding of the receptor or the mAB binding domain. Let *C*_*−*_ *, C*_*+*_denote the number of PAMs that lack or express CD163 on the cell surface, respectively, and *C*_*+−*_ denote the number of cells that were CD163 positive at one stage, but have shed the receptor or the mAB binding domain within the observation period. Thus *C*_*+*_ cells correspond to CD163 positive cells in the experimental observations, and *C*_*−*_ and *C*_*+−*_ cells together correspond to CD163 negative cells. It was assumed that *C*_*−*_ cells differentiate into *C*_*+*_cells at constant rate *d* and that *C*_*+*_ cells shed the receptor or the mAB binding domain at a rate *r*_*1*_ *.* To reproduce the observed increase in the proportion of cells classified as CD163 negative over the later incubation stages (Fig. 3b), it was assumed that all cells generate, at a constant rate *p*_*P*_, signalling molecules *P* with decay rate *s*_*P*_, and that these impact on receptor shedding in a cumulative, density dependent manner, as represented by the Michaelis-Menten function ${r}_1(P)=\frac{r_1P}{f_r+P}$, with asymptote *r*_1_ and the constant *f*_*r*_ denoting the half-saturation concentration. Non-infected cells were assumed to decay at constant rates *m*_*−*_ *, m*_*+*_ and *m*_*+−*_ *,* respectively. In model A, only *C*_*+*_ cells are susceptible to PRRSV. To accommodate the low susceptibility of PAMs at incubation day 0 observed in our experiment (Figs. 2c, d and 3a, c), which is a well-known artefact of in-vitro infection experiments with frozen PAMs and considered as an intrinsic property of the in-vitro environment [15], the infection rate *b(Q)* of *C*_*+*_ cells was also modelled as $b(Q)=\frac{b_{\mathtt{max}}Q}{f_b+Q}$ with maximum infection rate *b*_*max*_ and half saturation concentration *f*_*b*_ of compound *Q* which is assumed to be generated by the PAMs at a rate *p*_*Q*_ and to have decay rate *s*_*Q*_. The Michaelis-Menten function was chosen for the infection rate due to its mathematical properties allowing a fast rise to a constant maximum infection rate *b*_*max*_. Infected *C*_*+*_ (denoted *C*_+_^*^) cells were assumed to shed the receptor at rate ${r}_2(P)=\frac{r_2P}{f_r+P}$. Thus, if *r*_1_ ≠ *r*_2_ the shedding rate of CD163 differs between infected and non-infected cells. For example, infected cells may be more prone to shedding CD163 or the mAB binding domain than non-infected cells. Finally, infected cells can be either CD163 positive (*C*_+_^*^ or negative (*C*_+ −_^*^), with respective decay rates *a*_+_ and *a*_+−_.

The mathematical representation of Model A and the corresponding initial conditions are provided in the Additional file 4. Model parameters are listed in Additional file 5. Identifiability analysis revealed confounding between the rates of cellular processes depending on the compounds with the production and decay rates of these compounds. Therefore, production and decay rates were set to the arbitrary value of 0.5 in the model fitting process, which ensured identifiability of the remaining model parameters (see Additional file 2).

#### Hypothesis H2 and mathematical model B: CD163 is not essential for PRRSV infection. Instead, another (not yet identified) component controls PAM susceptibility to PRRSV, and CD163 may or may not be related to this

**Fig. 5:**
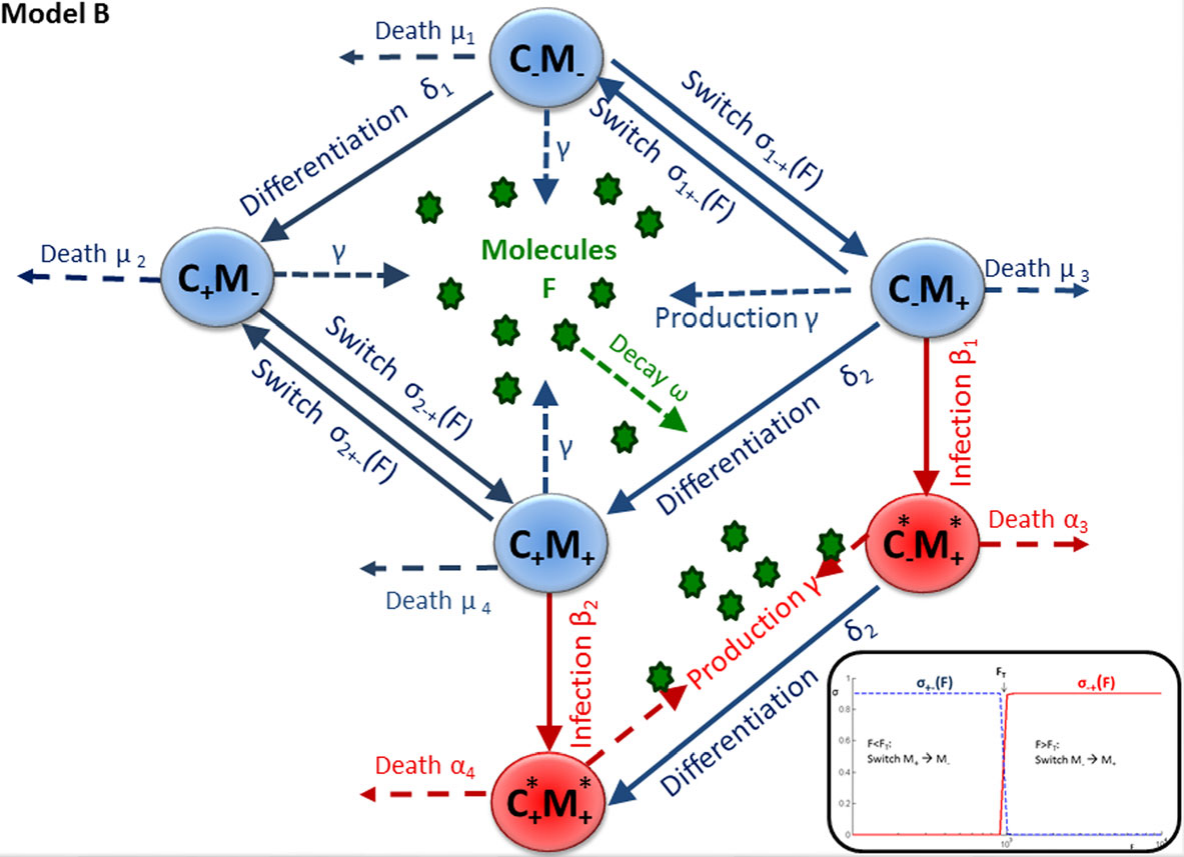
Schematic figure of model B. Components in *blue* and *green* refer to the cell types and biological processes represented at all stages of the experiment (i.e. prior and post infection with the PRRS virus or the mock agent). Components in red show additional cell types and processes after introduction of the virus into the cell cultures. *C*_*−*_ and *C*_*+*_ represent PAMs without/with the CD163 receptor on the cell surface, and *M*_*−*_ and *M*_*+*_ represent the non-susceptible and susceptible state, respectively. Cells indicated with ‘*’ denote infected cells. The bottom right panel illustrates the dependence of the switching rates σ_i+−_ and σ_i−+_ on the compound *F* (for parameter values *F*_*T*_ = 1000, ε = 0.1, σ_max_ = 0.9). See main text for more detailed description of the model, Additional file 4 for the mathematical representation of the model, and Additional file 5 for a description of the model parameters

The alternative hypothesis builds upon recent evidence that susceptibility of host cells to PRRSV may be more complex than conveyed by the susceptibility biomarker CD163 alone and may partly depend on other cellular entry mediators [32]. Indeed, we assessed the role of one such established susceptibility biomarker, i.e. CD169 (also known as Sialoadhesin or SIGLEC1) [18–20], in additional in vitro-experiments (see Additional file 6). However, the experimental results provide little support for a significant role of CD169 alone or combined with CD163 on the observed susceptibility trends (see Additional file 6). To our knowledge no other molecular marker that could explain the observed susceptibility trends has been identified to date. However, there is accumulating evidence that macrophage susceptibility to PRRSV or other viruses is characterised by a transient and reversible cellular state [15, 33, 34]. In line with these observations, we developed mathematical model B, in which host cell susceptibility was defined by a reversible cellular state ‘*M*’, which is controlled by components generated by the PAMs themselves (Fig. 5). In contrast to model A, model B assumes that CD163 is not essential for infection with PRRSV. In the absence of concrete evidence, the model incorporates both assumptions that expression of CD163 may or may not be related to the susceptibility state ‘*M*’. For example, CD163 may directly enhance switching into a susceptible state, or the development of CD163 may be regulated by the same components that render cells permissive to the virus.

Thus according to model B, prior to infection (or during mock infections) PAMs can be classed into four categories: CD163 negative and non-susceptible, CD163 positive and non-susceptible, CD163 negative and susceptible, and CD163 positive and susceptible. Let *C*_*−*_ *M*_*−*_ *, C*_*+*_ *M*_*−*_ *, C*_*−*_ *M*_*+*_ and *C*_*+*_ *M*_*+*_ denote the number of cells in these respective categories and *μ*_*1,*_ *μ*_*2,*_ *μ*_*2*,_ and *μ*_*4*_ their respective decay rates. *C*_*−*_ *M*_*−*_ cells differentiate into *C*_*+*_ *M*_*−*_ cells at constant rate *δ*_*1,*_ and *C*_*−*_ *M*_*+*_ differentiate into *C*_*+*_ *M*_*+*_ at a rate *δ*_*2.*_ Thus, *δ*_1_ ≠ *δ*_2_ would imply that differentiation from CD163 negative into CD163 positive cells depends on the susceptibility state *M*. Similar to model A, it is assumed that cells generate, at a constant rate γ, signalling molecules *F* (e.g. plasma proteins, transmembrane proteins, coagulation factors, enzymes or enzyme inhibitors) with decay rate ω that affect host cell permissiveness. Specifically, in model B, the density of these molecules in the medium is assumed to determine the susceptibility of PAMs to the virus. If the density of molecules *F* is low, susceptible M_+_ cells switch to a non-susceptible state at rate σ_1+−_(F) and σ_2+−_(F) for CD163 negative and CD163 positive cells, respectively. For high density F, non-susceptible M_−_ cells switch to a susceptible state (M_+_) at respective rates σ_1−+_(F) and σ_2−+_(F). The switching rates are represented by symmetric sigmoidal logistic functions:

$$ {\sigma}_{i-+}(F)={\sigma}_{i, max}\frac{1}{1+ \exp \left(-\varepsilon \left(F-{F}_T\right)\right)}\ \mathrm{and}\ {\sigma}_{i+-}(F)={\sigma}_{i, max}\left(1-\frac{1}{1+ \exp \left(-\varepsilon \left(F-{F}_T\right)\right)}\right) $$

where *i* = 1,2 represents CD163 negative and CD163 positive cells, respectively. Thus, as illustrated in the bottom right panel of Fig. 5, for F below the threshold F_T_, the rates σ_i−+_(F) of change from a non-susceptible to a susceptible state *M*_*−*_ to *M*_*+*_ are close to zero, whilst the rates σ_i−+_(F) from susceptible *M*_*+*_ to non-susceptible *M*_*−*_ are at their maximum *σ*_*i,max*_ *.* When *F* exceeds a threshold *F*_*T*_, the reverse happens: switching of cells from non-susceptible *M-* into susceptible state *M*_*+*_ occurs at maximum rates *σ*_*i* − +_(*F*) = *σ*_*i*,*max*_, whereas the opposite switch from susceptible to non-susceptible states occurs at rates close to zero. The constant *ε* determines how gradual the susceptibility state changes as *F* approaches *F*_*T*_. Note that *σ*_1,*max*_ ≠ *σ*_2,*max*_ corresponds to different switching rates for CD163 positive and negative cells.

In model B, only *M*_*+*_ cells are assumed susceptible to PRRSV. After introduction of PRRSV into the cultures at times T_i,_ (with subscript i representing different incubation times) *C*_*−*_ *M*_*+*_ cells become infected at rate *β*_*1*_ and *C*_*+*_ *M*_*+*_ cells become infected at rate *β*_*2.*_ Thus, *β*_2_ > *β*_1_ > 0 would imply that expression of CD163 on the cell surface is not essential, but enhances PAM susceptibility. Note that in contrast to model A, density dependent infection rates were not required in model B to reproduce the initial increase in cell permissiveness between the first incubation days, as susceptibility is controlled by the density dependent ‘*M*’ state in model B. Infected *C*_*−*_ *M*_+_* and *C*_*+*_ *M*_+_* cells decay at rates *α*_3_ and *α*_4_, respectively.

The mathematical representation of Model B and the corresponding initial conditions are provided in the Additional file 4. To ensure parameter identifiability production and decay rates were set to the arbitrary value of 0.5, and the parameter ε regulating the switching rate was set to the arbitrary value of 0.1 in model B for the model fitting (see Additional file 2). Thus, Model A contained 14 and model B contained 16 independent model parameters with unknown values (listed in Additional file 5).

### Inference of biological mechanisms underlying observed changes in cell susceptibility

#### The model results support hypothesis H2–CD163 was not essential for PRRSV infection

To infer which of the two proposed mathematical models has greater support from the experimental data, Models A and B were fitted to the data from the main in-vitro experiment as outlined in the Methods section. The computational algorithm used for model fitting led to a unique solution for all pigs for both models A and B, implying that in both cases the parameter values corresponding to the closet model fits to the data could be identified. Figure 2 shows that both models fit the majority of data reasonably well. However, the visual inspections (Fig. 2) and the model fit statistics (Table 1) provide an overwhelming support for model B, which not only produces a closer fit to the wide spectrum of kinetic profiles for all cell characteristics specified in the fitting criterion (Fig. 2), but also for the emerging properties (i.e. batch specific kinetic trends in susceptibility and CD163 expression) not directly included in the fitting criterion (Fig. 3). The superior fit of model B cannot be attributed to the greater number of parameters in model B, as the BIC criterion (Eq. 2) and the adjusted coefficient of determination *R*_*adj*_^2^ penalise over-parameterisation and a consistently superior fit was still achieved when model B was reduced to the same or even lower number of parameters than model A (by setting some parameters equal as outlined below and shown in Additional file 7).

**Table 1:** Comparison of the fits of mathematical models A and B to the experimental data

Pig (Batch)	Nr data points	RMS model A	RMS model B	BIC model A	BIC model B	*R*_*adj*_^2^ model A	*R*_*adj*_^2^ model B	Bias model A	Bias model B
1 (1)	25	0.088	0.026	−31.9	−58.4	0.87	0.99	0.071	0.005
2 (1)	25	0.068	0.024	−27.4	−55.8	0.91	0.98	0.003	0.055
3 (1)	25	0.070	0.036	−36.9	−46.1	0.92	0.97	−0.027	0.008
4 (2)	35	0.178	0.090	−29.4	−50.0	0.42	0.86	0.284	−0.035
5 (2)	35	0.135	0.048	−37.8	−69.1	0.43	0.92	0.276	−0.008
6 (3)	35	0.194	0.133	−26.7	−38.1	0.07	0.53	0.415	−0.033
7 (3)	35	0.196	0.111	−26.4	−43.8	0.11	0.73	0.689	−0.002
8 (3)	35	0.205	0.107	−25.1	−44.9	0.08	0.73	0.541	−0.034

RMS (Root Mean Square difference), BIC (Bayesian Information Criterion), *R*_*adj*_^2^ and total Bias were calculated according to Eqs. (1) to (4), respectively. Nr data points is the number of incubation periods for each experimental replicate (5 for batch 1 and 7 for batches 2 and 3) multiplied by five corresponding to the five fitting criteria ((i) the proportions of CD163 positive cells from the mock infected samples, and for the infected samples, the proportions of (ii) non-infected CD163 positive cells, (iii) non-infected CD163 negative cells, (iv) infected CD163 positive cells and (v) infected CD163 negative cells, respectively). For model B, equal switching rates for CD163 positive and CD163 negative cells (i.e. AS1 described below) were assumed as this produced the model of best fit (see section below)

Table 2 shows the parameter estimates for model B for each individual pig together with thestandard errors derived from the approximate covariance matrix of the estimate (Additional file 2). With a few exceptions, estimated parameter values associated with different pigs were of similar order of magnitude, and parameter estimates varied more between than within batches as would be expected due to genetic differences (Table 2). In cases where the parameter estimates differed substantially between pigs (e.g. the differentiation rate δ_1_) the standard errors were large, indicating a high degree of uncertainty in the parameter estimate.

**Table 2:** Estimated parameter values (with standard errors) for model B, assuming σ_1,max_ = σ_2,max_ = σ_max_

Pig	Pig 1	Pig 2	Pig 3	Pig 4	Pig 5	Pig 6	Pig 7	Pig 8
Batch (B)/Parameters	B1	B1	B1	B2	B2	B3	B3	B3
δ_1_	3.5E-5	2.4E-5	0.0027	0.0094	0.079	0.108	0.0633	1.0E-4
	(0.007)	(0.015)	(0.006)	(0.0075)	(0.011)	(0.020)	(0.002)	(0.003)
δ_2_	0.0041	0.053	0.049	0.0030	0.0061	0.011	0.0057	0.0047
	(0.001)	(0.034)	(0.006)	(0.0009)	(0.0017)	(0.011)	(0.021)	(0.024)
σ_max_	0.17	0.015	0.022	0.072	0.0056	1.0E-5	1.0E-9	5.1E-11
	(0.11)	(0.013)	(0.012)	(0.004)	(0.0089)	(0.0065)	(0.0012)	(0.0049)
F_T_	1.5E6	5.5E6	7.4E6	1.8E6	1.4E6	1.0E6	8.3E5	7.1E6
	(3.1E5)	(5.3E6)	(6.1E5)	(2.6E5)	(2.5E5)	(6.3E6)	(3.3E7)	(5.3E7)
μ_1_	0.0021	0.015	0.014	0.003	0.022	0.028	0.086	0.11
	(0.026)	(0.003)	(0.002)	(0.015)	(0.028)	(0.031)	(0.005)	(0.021)
μ_2_	0.144	0.036	0.031	0.15	0.20	0.135	0.14	0.11
	(0.0052)	(0.012)	(0.014)	(0.006)	(0.039)	(0.050)	(0.014)	(0.018)
μ_3_	4.9E-4	0.073	0.012	0.0030	3.5E-6	3.1E-7	1.0E-8	1.7E-10
	(6.5E-4)	(0.019)	(0.045)	(0.0015)	(5.7E-5)	(0.0017)	(0.023)	(0.026)
μ_4_	0.0070	0.014	0.0046	0.011	0.015	0.038	0.029	0.033
	(0.0015)	(0.052)	(0.014)	(0.0009)	(0.0049)	(0.022)	(0.020)	(0.014)
c_1_	0.00048	0.024	0.061	0.0089	0.0030	0.66	0.91	0.33
	(0.00044)	(0.13)	(0.170)	(0.023)	(0.0056)	(0.19)	(0.044)	(0.065)
c_2_	0.68	0.47	0.58	0.44	0.51	0.059	1.4E-5	0.58
	(0.05)	(0.44)	(0.063)	(0.058)	(0.11)	(0.12)	(0.040)	(0.037)
c_3_	0.099	0.50	0.36	0.34	0.22	0.040	0.012	0.011
	(0.008)	(0.19)	(0.48)	(0.015)	(0.010)	(0.012)	(0.004)	(0.003)
β_1_	0.94	0.99	1.00	0.54	0.31	0.20	0.24	0.21
	(0.10)	(2.89)	(1.50)	(0.030)	(0.019)	(0.022)	(0.015)	(0.044)
β_2_	0.76	0.18	1.00	1.00	0.40	0.29	0.31	0.37
	(0.025)	(0.18)	(0.54)	(0.042)	(0.070)	(0.12)	(0.054)	(0.080)
α_3_	0.63	0.98	1.00	0.53	0.77	0.40	0.36	0.31
	(0.38)	(0.03)	(0.04)	(0.0097)	(0.017)	(0.031)	(0.017)	(0.027)
α_4_	0.53	0.05	0.092	0.38	0.39	0.28	0.32	0.26
	(0.064)	(0.023)	(0.113)	(0.023)	(0.058)	(0.101)	(0.024)	(0.023)

Parameter values are given up to two significant digits. Standard errors were derived from the covariance matrix of the weighted least square estimates as described in Additional file 2

Table 3 shows the predictive abilities for models A and B as assessed by the *R*_*adj*_^2^ statistics, averaged over all individuals from the same batch in the predictor and validation set, respectively (e.g. the value associated with predictor set batch 1 and validation set batch 2 is the average *R*_*adj*_^2^ between individuals *l* and *j*, with individuals *l* from batch 1 and individuals *j* from batch 2). When predictions are within the same experimental batch (diagonal values in Table 3), model B has consistently considerably higher predictive ability than model A (average *R*_*adj*_^2^ for models B and A are 0.65 (sd = 0.22) and 0.34 (sd = 0.40), respectively). When prediction and validation sets refer to different batches, both mathematical model have no predictive ability (*R*_*adj*_^2^ = 0). This is expected as cells from different batches originate from different pig breeds that are likely to differ in PAM composition, susceptibility and transition rates represented in the models. However, it is important to point out that a poor predictive value of a model for specific fixed parameter values does not imply that the model provides a poor data fit for all parameter values, as was shown earlier (see e.g. Fig. 2). Similar results were found for the other statistics RMS and total Bias (see Additional file 8).

**Table 3:** Predictive ability of models A and B, assessed by the *R*_*adj*_^2^ statistics, averaged over all individuals from the predictor and validation batches

	Model A			Model B		
Validation set	Batch 1	Batch 2	Batch 3	Batch 1	Batch 2	Batch 3
Predictor set						
Batch 1	0.74	0	0	0.80	0	0
Batch 2	0	0.34	0	0	0.75	0
Batch 3	0	0	0.31	0	0	0.39

In summary, the in-vitro and in-silico results together point to CD163 not being essential for PRRSV infection in our experiment. Instead, the close fit of model B to the multi-dimensional data and the realistic and consistent parameter estimates provide support for the hypothesis that PAM susceptibility to PRRSV is defined by a reversible state that is regulated in a density dependent manner by signalling molecules or other unknown autocrine substances.

#### Relationship between CD163 and the inferred susceptibility state M

In principle, the parameter estimates obtained for model B can provide further insights into the nature of the inferred susceptibility state M and its relationship with the CD163 receptor. For example, consistently greater values for β_2_ than β_1_ would suggest that CD163 enhances susceptibility. However, caution is advised when interpreting individual parameter estimates as there is some degree of inter-dependence between different parameters (see collinearity indices in Additional file 2). Rather than inspecting individual parameter values we therefore tested whether simpler models consisting of fewer parameters would provide a similarly good fit by exploring the following assumptions:

1. The switching rate between the susceptibility states M_−_ and M_+_ is independent of CD163 (i.e. σ_1,max_ = σ_2,max_ = σ_max_)
2. PAM susceptibility to PRRSV is independent of CD163 (i.e. β_1_ = β_2_ = β)
3. The differentiation rate from a CD163 negative to CD163 positive state is independent of the susceptibility state M (i.e. δ_1_ = δ _2_ = δ)
4. The cellular decay rates are independent of CD163 and M (i.e. μ_1_ = μ_2_ = μ_3_ = μ_4_ = μ and α_3_ = α _4_ = α)

These assumptions were implemented into model B individually and in combination, and corresponding parameter estimates were obtained using the same fitting procedure as above. As the alternative models are nested, the log-likelihood ratio test could be applied to determine whether a particular model provides a significantly superior fit over another model. Based on the fit statistics, the model incorporating the first assumption (AS1) resulted in the model of best fit for all eight pigs. Compared to the full model B, the reduced model (AS1) produced a statistically significantly better fit than the original model for six out of eight pigs (*p* < 0.05). Thus, according to the model, the presence of CD163 has no direct effect on the rate at which cells switch between susceptible and non-susceptible states.

With regards to AS2, i.e. whether or not CD163 enhances susceptibility, the picture is less clear. According to the model fit statistics, the model allowing for CD163 dependent infection rates (β_1_ ≠ β_2_) provided a significantly superior fit over the model assuming no effect of CD163 on infection rates (β_1_ = β_2_) for only three out of the eight pigs (pigs 1, 4 and 5) (see Additional file 6). However, there was no systematic relationship between β_1_ and β_2_ that was common to all pigs and sensitivity estimates for some of the pig specific infection rates β_1_ and β_2_ were wide and overlapped for the majority of pigs (i.e. all except pigs 1 and 5) implying a large degree of uncertainty in these parameters (Table 2). Thus, the available data do not provide conclusive evidence on whether or not CD163 enhances susceptibility of PAMs to PRRSV.

Assumption AS3 resulted in significantly poorer model fits for all pigs, although the general trends were still captured reasonably well (Additional file 7). This would suggest that differentiation rates for CD163 are not independent of the cellular state M, as would also be supported by the distinct sensitivity intervals for the differentiation rates *δ*_1_ and *δ*_2_. Finally, assumption AS4 resulted in a substantially poorer model fit for all pigs (*p* < 0.05) indicating that mortality rates depend partly on CD163 and M. However, we could not detect a uniform pattern in the parameter estimates across all 8 pigs in support of the hypothesis that a particular cellular state has a systematic influence on either cell differentiation or longevity (Table 2).

#### Underlying causes for temporal changes in CD163 prevalence and in susceptibility to PRRSV

Figure 3 shows that model B reproduces the key characteristics of the in-vitro experiment, including the batch dependent non-linear trend in cell susceptibility to PRRSV (Fig. 3a, d), the consistent decline in the proportion of CD163 positive cells with time (Fig. 3b, e), and the decrease in tropism of the virus to CD163 positive cells over time (Fig. 3c, f). The mathematical model sheds some light on the potential causes for the observed heterogeneity between the experimental batches consisting of different pig breeds as differences in parameter estimates were generally larger between batches than between pigs within the same batch (Table 2). One striking difference between parameter estimates associated with batch 3 to those of batches 1 and 2 is the extremely low value for the maximum rate σ_max_ controlling the rate at which cells switch between non-susceptible (*M*_*−*_) and susceptible (*M*_*+*_ *)* states (Table 2). This would imply that samples from batch 3 changed their susceptibility state less frequently than samples from other batches. Since a relatively large proportion of cells in batch 3 were initially non-susceptible (i.e. *M*_*−*_ as indicated by *c*_1_ + *c*_2_ > 0.5, these parameter values would explain why for batch 3 susceptibility increased at a relatively slow rate, but remained high once cells had switched from the non-susceptible *M*_*−*_ to the susceptible *M*_*+*_ state (Fig. 3a, d). The apparent decline of CD163 positive cells within the subset of infected cells over increasing incubation time that was most pronounced in batch 3 (Fig. 3c, e) could be explained by the combination of two factors, i.e. the slow decline in the percentage of susceptible CD163 negative cells (due to relatively low decay rate *μ*_*3*_), and the fact that susceptibility is only partially controlled by CD163 in model B.

In summary, the model suggests that changes in PAM susceptibility to PRRSV over time are likely the result of several cellular processes interacting rather than caused by one single process alone. In particular, moderate differences in the initial composition of cells and in cell state dependent differentiation, activation and decay rates can generate a large inter-pig variation in kinetic susceptibility and biomarker trends, as observed in the in-vitro experiment.

## Discussion

Changes in host target cell susceptibility to an infectious agent can drastically alter the within-host infection dynamics and outcome of infection [35, 36]. However, dynamic changes in host cell composition throughout infection are difficult to inspect in vivo. Our in-vitro studies revealed that susceptibility of incubated PAMs to PRRSV can indeed change substantially over time, even in the absence of the virus and immune response. Surprisingly, the well-established susceptibility biomarker CD163 emerged as an unreliable indicator for change in cell susceptibility in our study. Indeed, our process-based models informed by the experimental data do not support the view that CD163 is essential for productive infection of PAMs with PRRSV [19–22]. Instead, models in which susceptibility is a reversible cellular state that is mediated in a density dependent manner by autocrine factors, provide a significantly superior fit to the multi-variate data than models imposing CD163 as essential component for susceptibility.

Mathematical models have proved a powerful tool to infer biological processes that are difficult to monitor experimentally [23, 24, 27, 36–38]. Validation of mathematical models requires however data of sufficient quality to discriminate between alternative possible model assumptions. The multi-dimensional longitudinal measures of various cell characteristics generated in our controlled laboratory environment proved sufficiently informative for discriminating between alternative hypotheses surrounding the role of CD163 in PRRSV infections. Indeed, model B (CD163 not essential for infection) unanimously produced a close and a statistically superior fit over alternative model A (CD163 essential for infection) to the multi-variate data profiles for all eight pigs, and led to realistic and consistent estimates for the model parameters. Our initial mathematical models also included the dynamics of the additional biomarker CD169, for which experimental measurements were collected, but for which double staining with virus antibodies was not possible in our experiment (see Additional file 6). The information obtained from this single staining alone proved insufficient for inferring the effect of CD169 on cell susceptibility, and this was clearly reflected by the lack of convergence of the fitting algorithm.

Convergence of the computational algorithm towards plausible parameter estimates and the corresponding tight model fit to the multi-variate data profiles provides statistical support that our mathematical model (i.e. refined model B) is a valid representation of the cell susceptibility and CD163 biomarker dynamics. Nevertheless, a tight model fit to experimental data alone does not prove that the model and all incorporated assumptions are correct. For example, without evidence for the contrary, we adopted the simple model assumption that the reversible susceptibility state is mediated in a density dependent manner by signalling molecules or other permissiveness altering substances that are produced by all cells at equal and constant rates. This is in line with the well-established fact that macrophages secrete a broad range of biologically active substances into their local milieu including enzymes; enzyme inhibitors; plasma proteins such as complement components, coagulation factors, and apolipoprotein E [39]. These factors regulate the functions of other cells such as interferon, interleukin 1, mitogens, and angiogenesis factor. The real process is likely to be more complex and may require the presence of other cellular receptors or compounds. For example, toll-like receptors (TLRs) are known to play an important role in pathogen recognition of host cells and the production of antiviral cytokines [40, 41]. However, devising more complex models unaccompanied by informative data would not produce relevant novel insights.

Although we sought to determine generic mechanisms influencing PAM infection dynamics that are common to all pigs, our mathematical models were fitted to data from each individual animal separately. This way we could incorporate differences between individuals or batches due to genetic or environmental factors by imposing minimal constraints on the model parameter values [38]. This approach allowed us to test whether our models are able to reproduce the observed between pig variation in the multi-variate temporal patterns and simultaneously generate common key characteristics such as the observed apparent increase in tropism of PRRSV towards CD163 negative cells.

Although our inference approach could not determine the exact relationship between CD163 expression and the reversible susceptibility state of PAMs, it indicates that these two characteristics may not be independent. This inter-dependence could cause confounding and potential difficulties for disentangling cause and effect in experimental data. Indeed, in experimental studies increased levels of infection are often correlated with increased expression of CD163 [21, 22, 42]. Furthermore, gain of function experiments report a strong relationship between CD163 expression and PAM susceptibility after 1 or 2 incubation days [19–22]. Our models however demonstrate that even if susceptibility was not directly controlled by CD163, the majority of infected cells could still be CD163 positive as a by-product of several interacting processes (Fig. 3d). Such inter-dependence between cell state (e.g. activation, maturation and polarization) and CD163 expression has been previously demonstrated [33, 43, 44].

It is long known that external stimuli for differentiation and reversible activation of porcine alveolar macrophages can alter their susceptibility to PRRSV [15]. In a similar experiment to ours (although with a different PRRSV type), Gaudreault et al. [8] also found an increase in PAM permissiveness to PRRSV within 3–4 incubation days and established that new mRNA synthesis of the cultured cells played an important role in this observed increase. The authors proposed that an anti-inflammatory cytokine environment might facilitate mRNA synthesis and thus infection [8]. Our model is in line with this hypothesis and would further suggest that mRNA synthesis depends on the cytokine environment in a dose dependent manner.

Accumulating evidence points to a critical role of macrophage polarization into classically activated (M1) or alternatively activated (M2) cells in the response of cells and immune components to pathogens [33, 34, 43, 44]. Recently, macrophage polarization has also been implicated in controlling PRRSV infection [45]. Polarization is transient and highly reversible [46]. It can be induced by diverse environmental stimuli, including cytokines produced by host cells as well as pathogens, and leads to different types of macrophage phenotype and function [34]. For example, it has been shown that polarization can alter macrophage susceptibility to HIV-1 infection [44]. Although little is known about the causal relationship between CD163 and macrophage polarization and resulting functions, polarization and expression of CD163 are clearly confounded. Alveolar macrophages from healthy mice and human lungs have been found to be predominantly M2 with high levels of expression of CD163 [44]. Some studies even consider CD163, together with other receptors and chemokines, as important determinants for discriminating between M1 and M2 polarization, as M2 cells generally express high levels of CD163 whereas M1 cells express low levels of this receptor [43]. Whether and how CD163 and macrophage polarization in combination affect mRNA synthesis and the permissiveness of PAMs to PRRSV is currently not known, but is reminiscent of previous work showing that PAMs isolated from different pig breeds harbouring different pro-inflammatory transcriptional states also differed in susceptibility to PRRSV [25, 26]. In summary, our model would clearly support the hypothesis of a cytokine mediated, transient and reversible macrophage polarization regulating the susceptibility of PAMs to PRRSV.

To our knowledge, this is the first study that elicits how PAM susceptibility to PRRSV can change over time in a closed restricted culture environment. Future studies with more diverse virus strains are needed to test the validity of our results for other PRRSV strains and in-vivo. Evidence suggests that different PRRSV strains may give rise to different cellular response mechanisms [47]. The in-vivo response is also likely to be mediated by many factors, including the virus, by-stander cells and immune components, which are not represented in the in-vitro system [13, 48, 49]. Furthermore, in vivo macrophages are likely to cover a continuous spectrum of activation phenotypes rather than two discrete polarized states as implied in our model [34], which may affect cell susceptibility and CD163 expression. Future studies using diverse PRRSV strains should therefore assess how PRRSV infection or specific immune components, rather than incubation, affects the susceptibility of resident non-infected macrophages and CD163 expression.

A recent in vivo gene editing experiment reports that pigs that lacked functional CD163 were fully resistant to a virulent North American PRRSV strain, with the virus unable to replicate in the alveolar macrophages of these pigs [50]. At first sight these findings appear contradictory to our findings. However, apart from obvious breed and virus strain differences, one fundamental discrepancy between this and our study is that all PAMs and precursor cells in the gene-edited pigs lacked functional CD163 through an edited mutation in exon 7. In contrast, PAMs in our study were derived from pigs with heterogeneous macrophage populations, where macrophages that did not express CD163 had the potential to differentiate into CD163 positive cells. One would expect that gene edited enforced manipulation of a particular CD163 pathway acting on cells of all maturity stages affects cell permissiveness to a virus differently than the natural cellular processes acting on incubated PAMs from wild-type pigs. Our study has therefore important implications for the development of vaccines or therapeutics that target CD163 expression in resident macrophages of pigs, as our findings would imply that simply reducing CD163 expression may not necessarily protect cells from infection with PRRSV. Clearly, further studies are needed to decipher the exact role of CD163 on host cell permissiveness to various PRRSV strains.

## Conclusions

The quantitative evidence produced by our dynamic model inference approach allowed us to discriminate between alternative hypotheses surrounding the underlying processes controlling dynamic changes in host cell susceptibility to an important virus. Using this approach we inferred that the susceptibility of alveolar macrophages is most likely a reversible state that may be mediated in a density dependent manner by compounds generated by the target cells themselves. The previously identified susceptibility bio-marker CD163 appears to have only played a secondary role in the observed infection dynamics. Macrophage polarization is one potential mechanism that has been shown to control host cell susceptibility to viruses and which would match the model inferred cell characteristics, but its role in infection dynamics related to PRRSV and other viruses still needs to be further established for the development of novel drug targets.

## Additional files

Additional file 1: Further information about the in vitro experiment. Description of the experimental protocol. (PDF 422 kb)
Additional file 2: Identifiability analysis. Description of the statistical identifiability analysis with corresponding results. (PDF 627 kb)
Additional file 3: Evidence from additional in-vitro experiments to test Hypothesis H1. Description of the experimental protocol and findings with regards to Hypotheses 1A–1C described in the main article. (PDF 445 kb)
Additional file 4: Mathematical representation of models A and B. Model equations, initial conditions and assumptions for statistical inference. (PDF 452 kb)
Additional file 5: List of parameters of the mathematical models A and B. Description of the parameters of the mathematical models A and B, together with information on whether these were assumed known or estimated by the fitting algorithm. (PDF 247 kb)
Additional file 6: Additional in-vitro experiments to assess kinetic trends in CD169 expression and the potential role of CD169 in mediating PAM susceptibility to PRRSV. Description of the experimental protocol and findings associated with CD169 expression of PAMs and their potential role in mediating PAM susceptibility to PRRSV. (PDF 491 kb)
Additional file 7: Comparison of fits of various refined versions of mathematical model B to the experimental data for eight pigs from 3 batches (B1–B3). The various versions of model B refer to different assumptions regarding the role of CD163 for PAM susceptibility. Black lines refer to the assumption AS1 in the main article (i.e. CD163 does not affect the rates of switching between M_−_ and M_+_, but directly enhances susceptibility of M_+_ cells to PRRSV (i.e. β_1_ < β_2_)). Blue lines represent the assumption AS2 (i.e. CD163 has neither direct nor indirect influence on PAM susceptibility). Green lines refer to assumption AS3 (i.e. the susceptibility state M has no influence on the differentiation rate from CD163 negative to CD163 positive state). Results for assumption AS4 in the main text are not plotted as they resulted in poor model fit and obstructed visibility of other model fits. Experimental data are represented by red circles. (A&B): percentage of non-infected PAMs classified as CD163 positive (A) and CD163 negative (B), respectively; (C&D): percentage of infected PAMs classified as CD163 positive (C) and CD163 negative (D), respectively. The fit statistics supported the assumption AS1 over AS2 (i.e. black lines fit better than blue lines; CD163 has no effect on the rate of switching between the susceptible and non-susceptible M states, but affects PAM susceptibility) for pigs 1, 4 and 5, but favoured AS2 over AS1 (blue lines over black lines) for the remaining pigs. Models incorporating AS3 (green lines) resulted in a significantly poorer model fit for all pigs than models representing AS1 and AS2 (black and blue lines). Models incorporating AS4 led to a significantly poorer model fit than those incorporating assumptions AS1─AS3 for all pigs. (TIF 273 kb)
Additional file 8: Additional tables to assess the predictive value of models A and B. Tables showing the predictive value of models A and B based on RMSE and total bias. (PDF 389 kb)

## Abbreviations

mAB: monoclonal antibody
PAMs: Porcine alveolar macrophages
PRRSV: Porcine Reproductive and Respiratory Syndrome Virus
