# Supplementary material for: Combining laboratory and mathematical models to infer mechanisms underlying kinetic changes in macrophage susceptibility to an RNA virus
Source: BMC Syst Biol. 2016 Oct 22;10:101. doi: 10.1186/s12918-016-0345-5 (PMC5075420; doi:10.1186/s12918-016-0345-5)
Supplement: Additional file 1: — Further information about the in vitro experiment. Description of the experimental protocol. (PDF 422 kb) [file 12918_2016_345_MOESM1_ESM.pdf]

Additional File 1: Further information about the in vitro experiment

Cell samples and preparation of alveolar macrophages by broncho-alveolar lavage

Three independent experimental batches of porcine alveolar macrophages (PAMs) were isolated from broncho-alveolar lung lavage (BALF) performed on eight 4-6 weeks old pigs originating from high health herds tested to be free of PRRSV, PCV2 and *Mycoplasma hypnomeumoniae* (3 Large White pigs in batch 1, 2 Large White -Landrace pigs in batch 2, and 3 Landrace-Yorkshire pigs in batch 3), as described previously [1]. PAMs, after recovery from frozen state, were cultured in RPMI 1640 Glutamax, containing 10%(v/v) FCS and 100 units/ml penicillin G and streptomycin sulphate (100ug/ml),(pen/strep). 12.5cm^2 flasks were seeded with 5 x 10^6 PAMs for varying lengths of time. Cells were harvested from the flasks after 15 minutes incubation at 4°C in growth medium containing 5mM EDTA prior to counting and staining. Screening of incubated cells with monocyte markers confirmed that over 99% of incubated cells were PAMs. The RPMI 1640 growth medium with GlutaMAX supplement, an alternative to L-glutamine with increased stability that improves cell health, and with the addition of vitamins, inositol and choline has been specially formulated to minimize toxic ammonia build-up and to maintain cell viability and growth. The growth medium was also supplemented with 10% Fetal Bovine Serum. RPMI 1640 medium uses a sodium bicarbonate buffer system (2.0 g/L) with 5–10% CO_2 environment to maintain physiological pH. Nevertheless, change in pH was monitored with Phenol Red. Measures obtained from the cell media at each incubation time indicated that pH, glucose and lactate concentration, and essential amino acids were stable within the 9 incubation days. Cell counts in cultures had dropped from to 5 x 10^6 cells at the beginning of incubation to about 10^5 cells after 9 incubation days. The viability of remaining cells in every culture replicate was above 80%, leaving a sufficient amount of viable cells in each culture to quantify changes in PAM susceptibility and CD163 expression. Because of decreasing cell numbers over time, the multiplicity of infection

varied between samples, but was consistently above three (i.e. between 3 and 6), implying that there were sufficient free viral particles to infect all susceptible cells, independent of the incubation stage. Furthermore, data used for the analyses refer to viable cells as only these were harvested from the bottom of the flasks.

Immunofluorescence assays

Cells were stained with the primary monoclonal antibodies mAB, 2A10/11–RPE (AbD serotec) (diluted as recommended by the manufacturer) for 60 minutes at room temperature (RT) and washed with PBS containing 2.5%(v/v) FCS (Invitrogen) and 0.01%(w/v) sodium azide (Sigma-Aldrich) (FACS/PBS). For each experiment, an appropriate isotype control was included to account for 'background' staining. Where unlabelled primary mAbs (CD169, see additional file 6) were used, cells were then stained with FITC-conjugated goat anti-mouse IgG(Fc specific) F(ab)2 fragment (Sigma-Aldrich), diluted 1/200 in FACS/PBS containing 3% (v/v) goat serum (Sigma-Aldrich), for 60 min at room temperature in the dark to detect bound mAbs. After washing, the fluorescent-labelled cells were re-suspended in Cell Wash (BD Biosciences) containing 0.4%(w/v) paraformaldehyde (Sigma-Aldrich) and were analysed by flow cytometry (CyAn ADP, DAKO). For the detection of PRRSV, fixed cells were washed in PBS containing 0.5%(w/v) saponin,(Sigma-Aldrich) 0.1%(w/v) bovine serum albumin (BSA) (Sigma-Aldrich), 0.01% (w/v) sodium azide (PBS/SAP) and permeabilised by resuspending in CaltagB (Invitrogen) containing 3%(v/v) mouse serum (Sigma-Aldrich). Primary mAb SDOW17-FITC (Rural Tech) was then added (1/100 diln) and incubated for 60 mins at RT in the dark. Cells were washed twice in PBS/SAP. The fluorescent-labelled cells were re-suspended and analysed by flow cytometry, using the Summit v 4.3 software. All dual stained samples were manually compensated to remove spectral overlap, using unstained and single positive stained controls to set the thresholds for cell classification (see black lines in Fig. A below). These controls helped to delineate and quantify positive and negative population and

represented the optimal approach to minimize group contamination with marginal error (of less than 1-3%) [2]. The data were orthogonalised for separation into distinct cell populations. Most importantly, this classification procedure was consistent across samples implying that potential misclassification of cells was consistent across sampling times and thus that observed temporal trends in cell characteristics were genuine.

Figure A shows an example for the flow cytometry results for PAMs from one of the pigs.

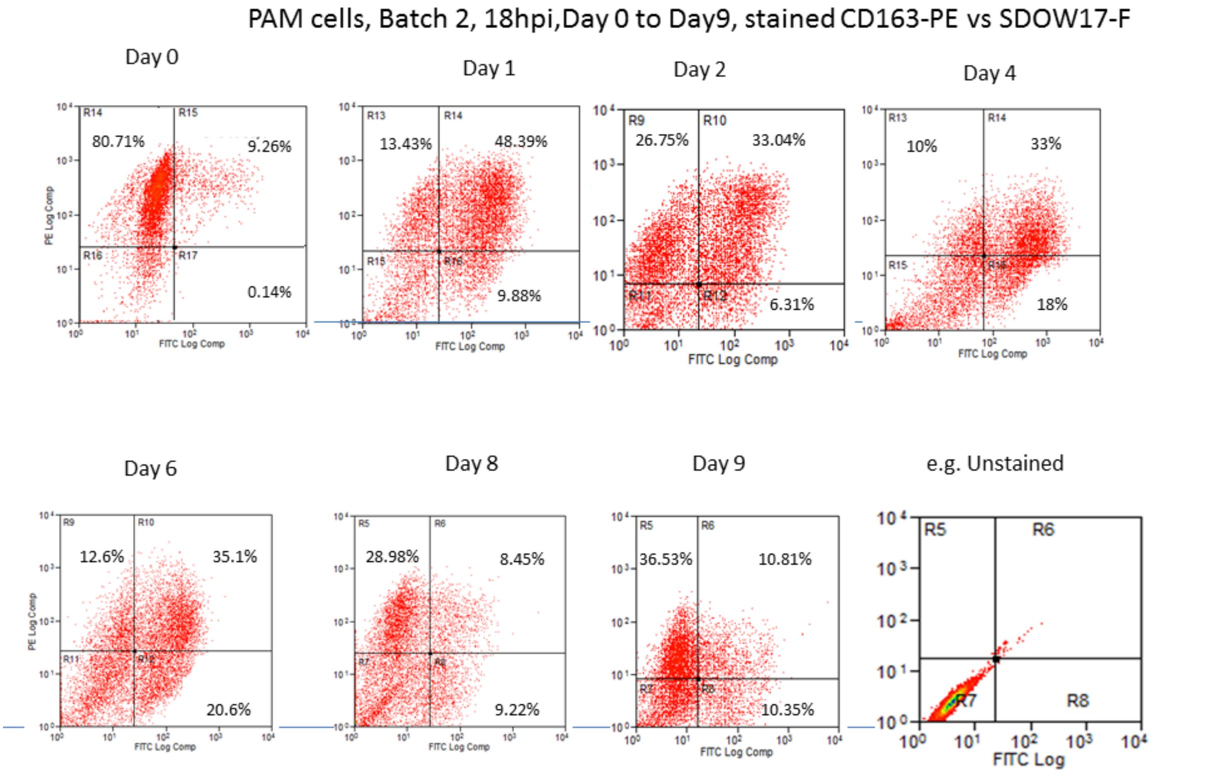

Figure A: Flow cytometry results for PAMs of one of the pigs from batch 2 after double staining of cells with CD163 antibody CD163-PE (y-axis) and PRRSV antibody SDOW17-F (x-axis) after 0, 1, 2, 4, 6, 8 and 9 days incubation and infection with PRRSV for 18 hours.

References:

[1] Ait-Ali T, Wilson AD, Westcott DG, Clapperton M, Waterfall M, Mellencamp MA et al. Innate immune responses to replication of porcine reproductive and respiratory syndrome virus in isolated Swine alveolar macrophages. Viral Immunol. 2007;20(1): 105-118.

[2] Herzenberg LA, Tung J, Moore WA, Herzenberg LA, Parks DR. Interpreting flow cytometry data: a guide for the perplexed. Nature immunology. 2006 Jul 1;7(7):681-5.
