# Supplementary material for: Combining laboratory and mathematical models to infer mechanisms underlying kinetic changes in macrophage susceptibility to an RNA virus
Source: BMC Syst Biol. 2016 Oct 22;10:101. doi: 10.1186/s12918-016-0345-5 (PMC5075420; doi:10.1186/s12918-016-0345-5)
Supplement: Additional file 2: — Identifiability analysis. Description of the statistical identifiability analysis with corresponding results. (PDF 627 kb) [file 12918_2016_345_MOESM2_ESM.pdf]

Additional File 2: Identifiability analysis

1. Statistical approach

Identifiability analysis was carried out for the mechanistic models A and B presented in the results section to diagnose and remedy poor identifiability of model parameters. Identifiability issues were diagnosed by lack of convergence in the parameter estimation processes and by inspecting collinearity indices of normalized sensitivity matrices established by Brun et al. [1] and outlined below. To remedy identifiability problems we adopted the widely used approach of partitioning parameters into subsets of parameters to be estimated from the data and subsets of parameters whose values were a priori fixed, ensuring that the remaining parameters are identifiable. The same parameter partitioning was applied to all pigs.

Identifiable parameter subsets were determined following the iterative approach outlined in Brun et al. [1], which consisted of calculating parameter importance indices for ranking parameters according to their importance and calculating collinearity indices associated with parameter subsets as described below. Parameter importance indices account for the sensitivity of model prediction to changes in individual parameter values, whereas collinearity indices account for the degree of near-linear dependence of sensitivity functions of parameter subsets [1]. For specified candidate parameter subsets, parameter estimation was then performed for each individual pig separately (but using the same subset of parameters to be estimated for all individuals) until collinearity indices reached a value below 20 [1] and convergence was achieved for every individual considered in this study.

For the statistical description of identifiability analysis, let $\boldsymbol{\hat{\theta}_{j,LS}} = (\hat{\theta}_{j1}, \dots, \hat{\theta}_{jm})$ denote the vector of weighted least estimates of model parameters for pig j, obtained by minimising the

residual sum of squares $RSS = \sum_{k=1}^{5}\sum_{i}^{n_j}(y_{jki} - \hat{y}_{jki})^2$, where y_{jki} refers to the i'th measurement of measure k, k=1, .., 5, for individual j, and $\hat{y}_{jki} = M_{ki}(\boldsymbol{\theta_j})$ are the corresponding predictions of model M with parameter vector $\boldsymbol{\theta_j} = (\theta_{j1}, \ldots, \theta_{jm})$. The (approximate) covariance matrix of the estimate $\widehat{\boldsymbol{\theta}}_{\boldsymbol{j,LS}}$ is defined as

$$\widehat{Var}[\widehat{\boldsymbol{\theta}}_{\boldsymbol{j,LS}}] = \frac{RSS_{min}}{n-m} \; (\boldsymbol{V}^{\boldsymbol{T}}\boldsymbol{V})^{-1}$$

with RSS_{min} being the minimum residual sum of squares, n and m being the number of observations (i.e. $5n_j$) and parameters, respectively, and **V** is the $n \times m$ derivative matrix $\mathbf{V} = \frac{\partial \boldsymbol{M}(\boldsymbol{\theta})}{\partial \boldsymbol{\theta}} \,|_{\boldsymbol{\theta} = \widehat{\boldsymbol{\theta}}_{j,LS}}$, calculated by finite difference approximation. Diagonal elements of the covariance matrix $\widehat{Var}[\widehat{\boldsymbol{\theta}}_{\boldsymbol{j,LS}}]$ were used to calculate standard errors for the model parameter estimates, whereas high off-diagonal elements indicated strong dependencies of parameter estimates and thus potential identifiability issues.

The sensitivity matrix **V** describes the sensitivity of the model output $\boldsymbol{M}(\boldsymbol{\theta})$ to small changes in the parameter values $\boldsymbol{\theta}$ at $\widehat{\boldsymbol{\theta}}_{\boldsymbol{j,LS}}$. To calculate parameter importance indices for ranking parameters according to their impact on model predictions, the scaled sensitivity matrix $\boldsymbol{S} = \{s_{ik}\}$ was calculated, with elements defined as

$$s_{ik} = v_{ik}\,\Delta\theta_k, \quad \text{i = 1, .., n and k = 1, …, m}$$

where v_{ik} denotes an element of the sensitivity matrix **V** and $\Delta\theta_k$ denotes the change in parameter θ_k (chosen here as $\Delta\theta_k = \theta_k$). Parameter importance indices were then derived by calculating the norms of the columns $\boldsymbol{s_k}$ of the **S**-matrix, given by $|s_k| = \sqrt{\frac{1}{n}\sum_{i=1}^{n} s_{ik}^2}$. A large norm $|s_k|$ implies that a change of $\Delta\theta_k$ in the parameter θ_k has an important effect on the model outcome vector. This makes the parameter identifiable with the data if all other parameters are fixed. For each pig, parameters were ranked based on their importance indices given by the norms $|s_k|$.

If identifiability issues were diagnosed with subsequent need for parameter subset selection, parameters with consistent lowest ranking across all pigs were allocated to the subset of parameters with a priori fixed values, and their values were fixed at arbitrary values. It is important to note that the estimates of the remaining parameters depend on the fixed values, implying a likely bias in the parameter estimates (as the fixed arbitrary value is different from the unknown true value). However, this potential bias does not prevent the identification of reasonable parameter values that adequately describe the data. Furthermore, it was neither the aim of this study nor would it be reasonable to expect to be able to identify true parameter values from the data.

To assess the identifiability of parameter subsets $\boldsymbol{K}$ consisting of k<m model parameters, the normalized sensitivity matrix $\tilde{\boldsymbol{S}}$ with columns $\tilde{\boldsymbol{s}}_l = \frac{s_l}{|s_l|}$, l = 1, ..m, was calculated and the collinearity index γ_K was calculated as $\gamma_K = \frac{1}{\sqrt{\lambda_k}}$, where λ_k denotes the smallest eigenvalue of $\tilde{\boldsymbol{S}}_K^T \tilde{\boldsymbol{S}}_K$ with $\tilde{S}_K$ being the $n \times k$ submatrix of $\tilde{S}$ containing only columns associated with the parameter subset $\boldsymbol{K}$ [1]. According to Brun et al. [1], the collinearity index γ_K has the following interpretation: "a change in the model output vector $\boldsymbol{M}(\boldsymbol{\theta})$ caused by a shift of a parameter $\theta_j \epsilon \boldsymbol{K}$ can be compensated, at least in the linear approximation, up to a fraction of 1 divided by the collinearity index γ_K by appropriate changes in the other parameters in the set $\boldsymbol{\theta_K}$. " In other words, a high collinearity index indicates that the parameter set is poorly identifiable. In this study a threshold of 20 was used for the collinearity index [1]; if a parameter set $\boldsymbol{K}$ resulted in a collinearity index below 20 for all pigs and the parameter estimation procedure converged, the parameter set was considered identifiable.

2. Results:

The full models A and B originally consisting of 16 and 19 parameters, respectively (see Additional File 5), led to identifiability problems with collinearity indices above 100.

Sensitivity analyses and parameter ranking based on the parameter importance indices described above revealed ambiguity in the parameters controlling the non-observed density dependent effects of autocrine substances (i.e. quantities P,Q in model A and F in model B), and confounding between the shedding and infection rates (f, Q_b in model A) and switching rates (F_T in model B) with the respective production rates (parameters p_P, p_Q in model A, and γ in model B) and decay rates (parameters s_P, s_Q in model A and ω in model B) of these compounds. To remedy this issue, we fixed all production and decay rates of these compounds to the arbitrary constant value of 0.5. For similar reasons, the constant ε that influences the switch in cell susceptibility in model B, was set to the arbitrary value of 0.1. The remaining set of parameters (14 and 16 for models A and B, respectively, shown in Table A) were estimated through model fitting.

Table A shows of parameter importance ranking for the final models A and B comprising 14 and 16 parameters to be estimated, respectively, for all pigs, together with the corresponding collinearity indices. Collinearity indices were below 21 for all pigs in both models. For both models, the parameter ranks were inconsistent across all pigs, but highly consistent between pigs from the same experimental batch (genetic line), indicating that between batch differences in the data are also reflected by differences in the relative importance of the diverse underlying processes.

References:

[1] Brun R, Reichert P, Künsch HR. Practical identifiability analysis of large environmental simulation models. Water Resources Research. 2001 Apr 1;37(4):1015-30.

Table A Parameter importance ranking and collinearity indices for the final models A and B, with the listed parameters estimated by model fitting.

Pig<br>Batch (B) / Parameters & collinearity index	Pig 1<br>B1	Pig 2<br>B1	Pig 3<br>B1	Pig 4<br>B2	Pig 5<br>B2	Pig 6<br>B3	Pig 7<br>B3	Pig 8<br>B3
**Model A**								
d	6	5	4	6	5	2	3	2
r_1	10	6	8	8	9	3	4	3
f_r	12	11	12	5	7	10	11	10
m_-	11	9	9	9	11	11	10	11
m_+	2	3	3	2	2	8	9	9
m_{+-}	3	2	2	3	3	1	1	1
c_1	1	1	1	1	1	9	7	8
c_2	7	8	11	12	12	5	8	7
b_{max}	4	4	5	4	4	4	2	4
f_b	12	11	12	5	7	10	11	10
r_2	8	7	6	7	6	6	5	5
a_+	5	10	13	11	13	7	6	6
a_-	9	12	7	13	10	12	12	12
**Collinearity index γ**	**13.4**	**15.1**	**19.8**	**11.4**	**18.3**	**13.0**	**4.6**	**12.9**
**Model B**								
δ_1	15	15	12	14	10	10	8	12
δ_2	9	5	3	12	9	9	11	11
σ_{max}	6	3	2	7	12	12	14	15
F_T	2	1	1	9	11	13	15	14
μ_1	3	2	5	10	13	11	10	10
μ_2	4	4	6	8	2	1	1	1
μ_3	12	10	15	13	15	15	13	13
μ_4	13	9	14	6	7	3	5	4
c_1	14	13	13	15	14	8	7	7
c_2	1	6	4	11	8	14	12	8
c_3	8	8	9	4	6	2	2	2
β_1	10	14	11	5	5	7	6	5
β_2	5	7	8	2	3	5	4	6
α_3	11	11	10	1	1	6	9	9
α_4	7	12	7	3	4	4	3	3
**Collinearity index γ**	**10.3**	**14.2**	**17.6**	**3.6**	**7.8**	**10.3**	**19.7**	**20.6**
