# Supplementary material for: Combining laboratory and mathematical models to infer mechanisms underlying kinetic changes in macrophage susceptibility to an RNA virus
Source: BMC Syst Biol. 2016 Oct 22;10:101. doi: 10.1186/s12918-016-0345-5 (PMC5075420; doi:10.1186/s12918-016-0345-5)
Supplement: Additional file 3: — Evidence from additional in-vitro experiments to test Hypothesis H1. Description of the experimental protocol and findings with regards to Hypotheses 1A–1C described in the main article. (PDF 445 kb) [file 12918_2016_345_MOESM3_ESM.pdf]

Additional File 3: Evidence from additional in-vitro experiments to test Hypothesis H1.

Hypothesis H1 assumes that CD163 is essential for PRRSV infection, but could not be detected by the mono-clonal antibodies used for CD163 detection. There are several ways in which CD163 could have escaped detection. These, together with the laboratory evidence in support or against these are stated below in Hypotheses H1a to H1c.

Hypothesis H1a: PAMs that did not express CD163 on the cell surface expressed CD163 internally, and were thus susceptible.

The mono-clonal antibodies used for CD163 detection in the main experiment bind to the receptors on the cell surface. However, PRRSV co-localises with CD163 at the early endosomes, with the Clathrin mediated endocytosis as the identified pathway of entry for PRRSV [39]. These findings imply that expression of CD163 inside the cell rather than on the cell surface is critical for permissiveness. To establish the relationship between internal and external cellular expression of CD163, the experiment was repeated for one of the replicates of batch 3 (for a restricted number of incubation days, i.e. day 2 and day 6, due to a limited number of available cells) using a permeable staining method that allowed detection of both cell surface and cytosolic CD163 (Fig. A). However, the proportion of cells classified as CD163 positive by either method was similar (Table A), suggesting that PAMs classified as CD163 negative did not escape detection due to internal expression of the receptor. Based on this evidence, hypothesis H1a was rejected and not considered in the subsequent modelling studies.

Fig A. Confocal image produced by staining of PAMs with monoclonal antibody mAB 2A10 labeled with FITC (AbD Serotec) after permeabilization (PBS 0.1% Triton X100; Sigma-Aldrich) to detect cell surface (External) and cytosolic (Internal) CD163.

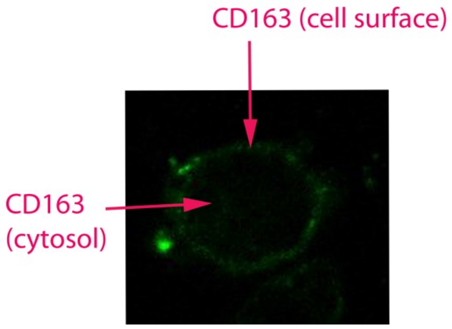

Stained cells were mounted using Vectashield medium containing DAPI (Vector Laboratories, UK). Confocal images were obtained using a Zeiss LSM710 inverted confocal microscope and analysed using Zen2011 software (Carl Zeiss, UK).

Table A. Percentage (mean percentage and standard deviation for mock infected samples) of PAMs classified as CD163 positive post PRRSV and mock infection, respectively, using two types of staining methods to screen for expression of CD163 on the cell surface (External) and inside the cell (Internal).

Day	Infection Type	%CD163 positive External	%CD163 positive Internal	%CD163 positive within infected cells (External)	%CD163 positive within infected cells (Internal)
**2**	Mock	51.5 (0.3)	56.0 (1.2)	NA	NA
	PRRSV	45.16	43.2	66.9	46.5
**6**	Mock	30.7 (1.8)	44.0 (8.7)	NA	NA
	PRRSV	4.0	4.0	7.2	7.1

In the main experiment the conventional External method was used. Results refer to additional available cells from one of the individuals from batch 3 in the main experiment (1 replicate for PRRSV infected samples and 3 replicates for mock infected samples).

Hypothesis H1b: CD163 changed its functional form throughout incubation and could therefore not be detected by the mono-clonal antibodies

The monoclonal antibodies (mAB 2A10) used for CD163 detection in the main *in-vitro* experiment of this study adhered to the standard screening protocol for CD163 in *in-vitro* cultures (e.g. Sanchez et al., 1999; van Gorp et al. 2010). However, if the receptor underwent functional form changes in the antibody binding domain throughout incubation or in response to the virus, PAMs expressing CD163 may have been erroneously classified as CD163 negative. To test this hypothesis, a different monoclonal antibody (denoted here AB_2; R and D systems, Cat No mAB AF1607) that binds to different regions of CD163 (Fig. B) was applied to a subset of cells from batch 3 (i.e. PAMs from an additional animal that was not used in the main experiment due to insufficient cell numbers). Replicates of mock and PRRSV infected cell cultures were set up for 2, 6 and 8 incubation days, respectively, and CD163 detection was carried out either with the original monoclonal antibody (mAB_1) or with mAB_2. Staining with mAB_2 consistently led to a higher percentage of cells classed as CD163 positive (Table B). However, the trends in PAM susceptibility and bio-marker kinetics were similar for both types of antibodies. In particular, the previously observed increasing tropism of PRRSV towards CD163 negative cells at later incubation stages was confirmed for both types of antibodies (Table B). As the different antibody types cover alternative regions of CD163 (Fig. B), it is unlikely that the high proportions of infected cells classified as CD163 negative in the later incubation stages of the main experiment were indeed CD163 positive. Based on these results, Hypothesis H1b was rejected and not considered in the subsequent modelling studies.

Fig B. Schematic representation of CD163 on the cell surface and of the domains that are recognized by mAB$_1$ (mAb 2A10; AbD Serotec) or mAB$_2$ (mAB AF1607; R&D Systems).

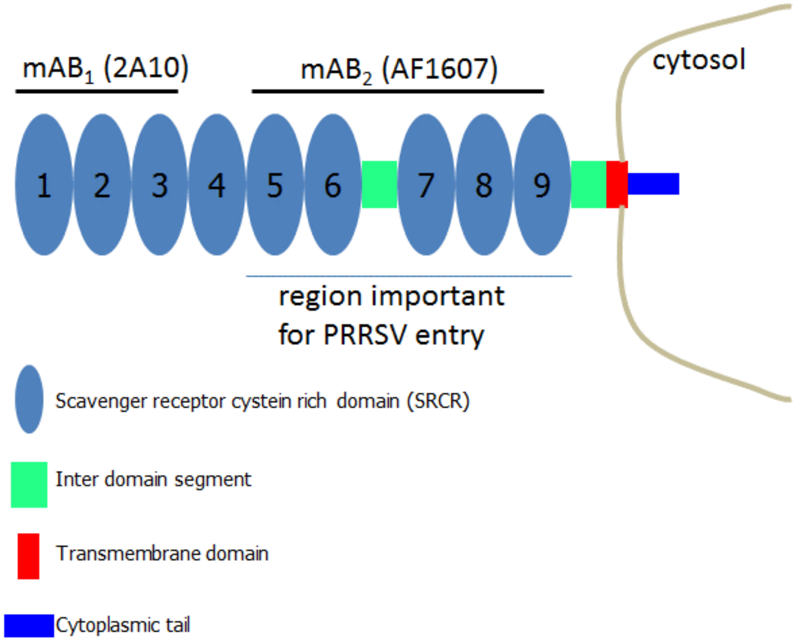

The region important for PRRSV entry is also shown.

Table B. Mean percentage with standard errors (in brackets) of PAMs classified as CD163 positive post PRRSV and mock infection, respectively, using two types of antibodies with alternative binding domains (mAB$_1$ and mAB$_2$).

**Day**	**Infection Type**	**%CD163 positive mAB$_1$**	**%CD163 positive mAB$_2$**	**%CD163 positive within infected cells (mAB$_1$)**	**%CD163 positive within infected cells (mAB$_2$)**
**2**	Mock	73.8	87.9	NA	NA
	PRRSV	45.6 (1.1)	75.6 (0.05)	49.4 (1.4)	78.3 (0.1)
**6**	Mock	53.8	80.3	NA	NA
	PRRSV	11.2 (4.8)	31.0 (3.1)	17.8 (8.2)	44.1 (5.9)
**8**	Mock	63.7	78.6	NA	NA
	PRRSV	5.2 (0.3)	23.3 (1.0)	7.2 (0.8)	30.9 (2.0)

In the main experiment mAB$_1$ was used. Results refer to cells from two and one pig (1 replicate per pig) from batch 3 for the PRRSV infected samples and mock infected samples, respectively.

Hypothesis H1c: PAMs classified as CD163 negative 18 hpi expressed the receptor at the time of infection and shed or changed functional form of the receptor within the 18h infection period, thus escaping detection

Evidence suggests that CD163 is not only expressed as a membrane bound protein but can also be actively shed from the cell surface to produce soluble CD163 (e.g. Fabriek et al., 2005). Furthermore, it cannot be excluded that in our experiment CD163 changed its functional form during the 18h period between (mock) infection and cell screening and thus escaped detection at the observation time (H. Nauwynck, pers. communic.). To determine the extent of CD163 shedding or form change in our system, additional cell cultures were set up for PAMs of two of the pigs from batch 3 for incubation days 2, 6 and 8, respectively, in which the percentage of cells expressing CD163 at the cell surface was measured at 0 hours rather than at 18 hpi as in the original samples. The results of this small scale experiment show that the percentage of CD163 positive cells was significantly lower at 18 hpi compared to 0 hpi (Table C), thus supporting the above hypothesis. Furthermore, the differences were more pronounced in the infected than in the mock infected samples, and at later incubation stages, indicating that both PRRSV infection and cell maturation may promote receptor shedding or form change.

Table C. Mean percentage with standard errors of PAMs expressing CD163 on the cell surface at 0 hours and 18 hours post PRRSV and mock infection, respectively, for 2 replicates of batch 3.

**Incubation day**	**%CD163 positive cells prior to (mock) infection**	**%CD163 positive cells 18 hours post PRRSV infection**	**%CD163 positive cells 18 hours post mock infection**
**2**	58.3 (9.4)	46.8 (1.7)	63.5 (11.9)
**6**	53.7 (18.1)	2.6 (1.4)	41.8 (11.1)
**8**	58.2 (NA)	0.6 (NA)	43.7 (NA)

For incubation day 8, only one replicate was available.
