# Supplementary material for: Combining laboratory and mathematical models to infer mechanisms underlying kinetic changes in macrophage susceptibility to an RNA virus
Source: BMC Syst Biol. 2016 Oct 22;10:101. doi: 10.1186/s12918-016-0345-5 (PMC5075420; doi:10.1186/s12918-016-0345-5)
Supplement: Additional file 4: — Mathematical representation of models A and B. Model equations, initial conditions and assumptions for statistical inference. (PDF 452 kb) [file 12918_2016_345_MOESM4_ESM.pdf]

**Additional File 4: Mathematical equations for models A and B, and assumptions for statistical inference.**

This document shows the mathematical equations for models A and B in the main document. We refer to the main document for the model description and to Figures 3 and 4 for schematic illustrations of models A and B, respectively. A description of the model parameters can be found in Additional File 4.

**ODE systems for Model A (representing hypothesis H1): CD163 is essential for PRRSV infection of PAMs**

**(a) Prior to introduction of the virus or throughout mock infection**

In order to distinguish between the system prior and post introduction of the PRRS virus, cell numbers referring to the system prior to infection or during mock infection are denoted by superscript $^0$.

$$
\begin{aligned}
\frac{dC_-^0}{dt} &= -(m_- + d)C_-^0 \\
\frac{dC_+^0}{dt} &= d\,C_-^0 - (m_+ + r_1(P^0))C_+^0 \\
\frac{dC_{+-}^0}{dt} &= r_1(P^0)C_+^0 - m_{+-}\,C_{+-}^0 \qquad\qquad \text{(A1)} \\
\frac{dP^0}{dt} &= p_P(\,C_-^0 + \ C_+^0 + \ C_{+-}^0) - \ s_P P^0 \\
\frac{dQ^0}{dt} &= p_Q(\,C_-^0 + \ C_+^0 + \ C_{+-}^0) - \ s_Q Q^0
\end{aligned}
$$

Where the shedding rate $r_1(P)$ is represented by the Michaelis-Menten function $r_1(P) = \frac{r_1\,P}{f_r+P}$ with asymptote $r_1$ and half-saturation concentration $f_r$.

The initial conditions (corresponding to t = 0) are:

$$C_-^0(0) = c_1 N_0\ ,\ C_+^0(0) = c_2 N_0,\ C_{+-}^0(0) = (1 - c_1 - c_2) N_0$$

$$\text{and } P^0(0) = Q^0(0) = 0. \qquad \text{(ICA1)}$$

where $N_0$ refers to the initial cell number at incubation, and $c_1$ and $c_2$ denote the proportions of $C_-$ and $C_+$ cells at the time of incubation, respectively. It was assumed that $N_0 = 5 * 10^6$ in all cell cultures.

System (A1) with initial conditions (ICA1) was used to model the cellular processes in the in-vitro system from incubation (t=0) to times $T_i = 4 + i \times 24\ [hours]$, with $i = 0, 1, 2, 4, 6, 8\ or\ 9$, for the replicates that were infected with the PRRS virus at times $T_i$, and from incubation to times $T_i$ + 18 hours for the mock-infected replicates.

**(b) Post introduction of the virus at incubation times Ti:**

In the in-vitro experiment, the virus was introduced into the cultures at incubation times $T_i = 4 + i \times 24$ hours, where $i = 0, 1, 2, 4, 6, 8$ or 9[days]. Thus, between the time intervals [$T_i$, $T_i$ +18] hours, the mathematical model for the cellular dynamics in the infected replicates is:

$$\frac{dC_-}{dt} = -(m_- + d)C_-$$

$$\frac{dC_+}{dt} = d\, C_- - (m_+ + r_1(P) + b(Q))C_+$$

$$\frac{dC_{+-}}{dt} = r_1(P)C_+ - m_{+-}\, C_{+-}$$

$$\frac{dC_{+}^{*}}{dt} = b(Q)C_{+} - (r_2(P) + a_{+})C_{+}^{*} \quad \text{(A2)}$$

$$\frac{dC_{+-}^{*}}{dt} = r_2(P)C_{+}^{*} - a_{+-}\, C_{+-}^{*}$$

$$\frac{dP}{dt} = p_P\,(C_{-} + C_{+} + C_{+-} + C_{+}^{*} + C_{+-}^{*}) - s_P P$$

$$\frac{dQ}{dt} = p_Q\,(C_{-} + C_{+} + C_{+-} + C_{+}^{*} + C_{+-}^{*}) - s_Q Q$$

Where the shedding rate $r_2(P)$ is represented by the Michaelis-Menten function $r_2(P) = \frac{r_2\, P}{f+P}$ with asymptote $r_2$ and the infection rate $b(Q)$ is represented by $b(Q) = \frac{b_{max}\, Q}{f_b+Q}$ with asymptote $b_{max}$ and half-saturation concentration $f_b$.

The initial conditions are:

$C_{-}(T_i) = C_{-}^{0}(T_i),\ C_{+}(T_i) = C_{+}^{0}(T_i),\ C_{+-}(T_i) = C_{+-}^{0}(T_i),$

and $C_{+}^{*}\,(T_i) = 0, C_{+-}^{*}\,(T_i) = 0,\ \mathrm{P}(T_i) = P^{0}(T_i),\ \mathrm{Q}(T_i) = Q^{0}(T_i).$ (ICA2)

**ODE systems for Model B (representing hypothesis H2): CD163 is not essential for PRRSV infection of PAMs**

**(a) Prior to introduction of the virus or throughout mock infection**

Similar to model A, cell numbers referring to the system prior to infection or during mock infection are denoted by superscript 0.

$$\frac{d(C_-^0 M_-^0)}{dt} = -\left(\delta_1 + \mu_1 + \sigma_{1-+}(F^0)\right) C_-^0 M_-^0 + \sigma_{1+-}(F^0)\, C_-^0 M_+^0$$

$$\frac{d(C_+^0 M_-^0)}{dt} = \delta_1\, C_-^0 M_-^0 - (\mu_2 + \sigma_{2-+}(F^0)\,)\, C_+^0 M_-^0 + \sigma_{2+-}(F^0)\; C_+^0 M_+^0$$

$$\frac{d(C_-^0 M_+^0)}{dt} = \sigma_{1-+}(F^0)\; C_-^0 M_-^0 - (\,\sigma_{1+-}(F^0) + \delta_2 + \mu_3)\, C_-^0 M_+^0 \qquad \text{(B1)}$$

$$\frac{d(C_+^0 M_+^0)}{dt} = \sigma_{2-+}(F^0)\, C_+^0 M_-^0 + \delta_2\, C_-^0 M_+^0 - \left(\mu_4 + \sigma_{2+-}(F^0)\right) C_+^0 M_+^0$$

$$\frac{dF^0}{dt} = \gamma\,(\,C_-^0 M_-^0 + C_+^0 M_-^0 + C_-^0 M_+^0 + C_+^0 M_+^0) - \omega F^0$$

With initial conditions (corresponding to t=0):

$$C_-^0 M_-^0(0) = \rho_1 N_0\,,\; C_+^0 M_-^0(0) = \rho_2 N_0,\; C_-^0 M_+^0(0) = \rho_3\, N_0,$$

$$C_+^0 M_+^0(0) = (1 - \rho_1 - \rho_2 - \rho_3) N_0, \qquad \text{(ICB1)}$$

$$F^0(0) = 0.$$

Where $N_0$ refers to the initial cell number at incubation ($N_0 = 5 * 10^6$), and $\rho_1$, $\rho_2$ and $\rho_3$ denote the proportions of $C_-M_-$ , $C_+M_-$ and $C_-M_+$ cells at the time of incubation, respectively.

The switching rates $\sigma_{i-+}(F)$ and $\sigma_{i+-}(F)$ , $i = 1,2,$ are represented by the logistic functions:

$$\sigma_{i-+}(F) = \sigma_{i,max} \frac{1}{1+\exp(-0.1(F-F_T))} \text{ and } \sigma_{i+-}(F) = \sigma_{i,max} \left(1 - \frac{1}{1+\exp(-0.1(F-F_T))}\right).$$

**(b) Post introduction of PRRSV at incubation times $T_i$:**

The dynamic processes post infection at incubation times $T_i = 4 + i \times 24$ $[hours]$, with $i = 0, 1, 2, 4, 6, 8$ $or$ $9$, are described by the following ODE system:

$$\frac{d(C_-M_-)}{dt} = -(\delta_1 + \mu_1 + \sigma_{1-+}(F))C_-M_- + \sigma_{1+-}(F)C_-M_+$$

$$\frac{d(C_+M_-)}{dt} = \delta_1 C_-M_- - (\mu_2 + \sigma_{2-+}(F))C_+M_- + \sigma_{2+-}(F)\, C_+M_+$$

$$\frac{d(C_-M_+)}{dt} = \sigma_{1-+}(F)\, C_-M_- - (\sigma_{1+-}(F) + \delta_2 + \mu_3 + \beta_1)C_-M_+$$

$$\frac{d(C_+M_+)}{dt} = \sigma_{2-+}(F)C_+M_- + \delta_2\, C_-M_+ - (\mu_4 + \sigma_{2+-}(F) + \beta_2)C_+M_+ \qquad \text{(B2)}$$

$$\frac{d(C_-^*M_+^*)}{dt} = \beta_1\, C_-M_+ - \alpha_3 C_-^*M_+^*$$

$$\frac{d(C_+^*M_+^*)}{dt} = \beta_2\, C_+M_+ - \alpha_4 C_+^*M_+^*$$

$$\frac{dF}{dt} = \gamma\,(C_-M_- + C_+M_- + C_-M_+ + C_+M_+ + C_-^*M_+^* + C_+^*M_+^*) - \omega F$$

With initial conditions given by the corresponding cell / signalling molecule numbers at the time of infection, i.e.

$$C_-M_-(T_i) = C_-^0M_-^0(T_i),\ C_+M_-(T_i) = C_+^0M_-^0(T_i),\ C_-M_+(T_i) = C_-^0M_+^0(T_i),$$

$$C_+M_+(T_i) = C_+^0M_+^0(T_i),\ C_-^*M_+^*(T_i) = 0,\ C_+^*M_+^*(T_i) = 0,\ \mathrm{F}(T_i) = F^0(T_i\ ).\ \text{(ICB2)}$$

**Using models A and B for statistical inference of dynamic processes underlying observed trends in host cell susceptibility**

To infer which of the two proposed mathematical models representing contrasting hypothesis about the importance of CD163 for PAM susceptibility to PRRSV has greater support from the experimental data, Models A and B were fitted to the data from the main *in-vitro* experiment as outlined in the Material and Methods section. Models A and B both include cellular processes that depend on the presence of non-observed compounds with specific production and decay rates. As the rates of cellular processes depending on the compounds are confounded with the production and decay rates of these compounds, production and decay rates were set to the arbitrary value of 0.5. Also, without loss of generality and to accelerate convergence, the constant ε in model B was set to 0.1, Thus, Model A contained 14 and model B contained 16 independent model parameters with unknown values (see Additional File 5).
