# Supplementary material for: Combining laboratory and mathematical models to infer mechanisms underlying kinetic changes in macrophage susceptibility to an RNA virus
Source: BMC Syst Biol. 2016 Oct 22;10:101. doi: 10.1186/s12918-016-0345-5 (PMC5075420; doi:10.1186/s12918-016-0345-5)
Supplement: Additional file 5: — List of parameters of the mathematical models A and B. Description of the parameters of the mathematical models A and B, together with information on whether these were assumed known or estimated by the fitting algorithm. (PDF 247 kb) [file 12918_2016_345_MOESM5_ESM.pdf]

Additional File 5: List of parameters of the mathematical models A and B, respectively.

Symbol	Definition	Unit	Value
**MODEL A**			
d	Differentiation rate from C_- to C_+	[1/h]	estimated
r_1	Maximum shedding rate for non-infected CD163 positive cells, i.e. transition rate from C_+ to C_{+-}	[1/h]	estimated
f_r	Half saturation concentration of compound P for receptor shedding	[1]	estimated
m_-, m_+, m_{+-}	Mortality rates for non-infected C_-, C_+ and C_{+-} cells, respectively	[1/h]	estimated
c_1, c_2	Proportion of C_- and C_+ cells, respectively, at start of incubation (time = 0)	[1]	estimated
b_{max}	Maximum infection rate	[1/h]	estimated
f_b	Half saturation concentration of compound Q for infection	[1]	estimated
p_P, p_Q	Production rate of compounds P and Q, respectively	[1/h]	0.5*
s_P, s_Q	Decay rates of compounds P and Q, respectively	[1/h]	0.5*
r_2	Maximum shedding rate for infected CD163 positive cells, i.e. transition rate from C^*_+ to C^*_{+-}	[1/h]	estimated
a_+, a_-	Mortality rates for infected C^*_+ and C^*_{+-} cells, respectively	[1/h]	estimated
**MODEL B**			
δ_1	Differentiation rate from C_-M_- to C_+M_-	[1/h]	estimated
δ_2	Differentiation rate from C_-M_+ to C_+M_+	[1/h]	estimated
$\sigma_{1,max}$	Max. (de) activation rate between C_-M_- and C_-M_+	[1/h]	estimated
$\sigma_{2,max}$	Max. (de) activation rate between C_+M_- and C_+M_+	[1/h]	estimated

ε	Constant determining how gradual the susceptibility state switches as F approaches F_T	[1]	0.1*
F_T	Threshold value for compound F	[1]	estimated
γ	Production rate for compound F	[1/h]	0.5*
ω	Decay rate for compound F	[1/h]	0.5*
$\mu_1, \mu_2, \mu_3, \mu_4$	Mortality rates for non-infected C_-M_-, C_+M_-, C_-M_+ and C_+M_+ cells, respectively	[1/h]	estimated
$\lambda_1, \lambda_2, \lambda_3$	Proportion of C_-M_-, C_+M_- and C_-M_+ cells, respectively, at start of incubation (t=0)	[1]	estimated
β_1	Infection rate for C_-M_+ cells, i.e. transition rate from C_-M_+ to $C_-^*M_+^*$	[1/h]	estimated
β_2	Infection rate for C_-M_+ cells, i.e. transition rate from C_+M_+ to $C_+^*M_+^*$	[1/h]	estimated
α_3, α_4	Mortality rates for infected $C_-^*M_+^*$ and $C_+^*M_+^*$ cells, respectively	[1/h]	estimated

Identifiability analysis revealed poor identifiability of model parameters controlling the density dependent effects of autocrine substances (i.e. quantities P,Q in model A and F in model B) and confounding between the shedding and infection rates (f, Q_b in model A) and switching rates (F_T in model B) with the respective production rates (parameters p_P, p_Q in model A, and γ in model B) and decay rates (parameters s_P, s_Q in model A and ω in model B) of these compounds. To remedy this issue, we fixed all production and decay rates of these compounds to the arbitrary constant value of 0.5 and estimated the remaining parameters through model fitting. For similar reasons, the constant ε in model B was set to the arbitrary value of 0.1.
