# Supplementary material for: Combining laboratory and mathematical models to infer mechanisms underlying kinetic changes in macrophage susceptibility to an RNA virus
Source: BMC Syst Biol. 2016 Oct 22;10:101. doi: 10.1186/s12918-016-0345-5 (PMC5075420; doi:10.1186/s12918-016-0345-5)
Supplement: Additional file 6: — Additional in-vitro experiments to assess kinetic trends in CD169 expression and the potential role of CD169 in mediating PAM susceptibility to PRRSV. Description of the experimental protocol and findings associated with CD169 expression of PAMs and their potential role in mediating PAM susceptibility to PRRSV. (PDF 491 kb) [file 12918_2016_345_MOESM6_ESM.pdf]

**Additional File 6:**

**Additional in-vitro experiments to assess kinetic trends in CD169 expression and the potential role of CD169 in mediating PAM susceptibility to PRRSV.**

**Objectives and Methods:**

Additional replicates were set up using PAMs of the 5 pigs from the experimental batches 2 (pigs 4 and 5 in the main article) and 3 (pigs 6 to 8 in the main article) to monitor kinetic trends of CD169 (also known as Sialoadhesin and SIGLEC1) marker dynamics over incubation time, in addition to the susceptibility and CD163 expression trends described in the main article. The additional cultures were subjected to same experimental protocol as the cultures described in the main article. In other words, PAMs from each pig were assigned to one of two different culture replicates with one replicate subjected to infection with the European type I PRRSV strain and the other replicate subjected to mock-infection with the equivalent volume of growth medium. To assess changes in CD169 expression, cells in each replicate were then distributed into separate cultures each comprising 5 x $10^6$ cells with corresponding incubation periods of 0, 1, 2, 4, 6, 8 and 9 days (or, more precisely, for 4, 28, 52, 100, 148, 196 and 220 hours post extraction from freezer), respectively, before either PRRSV or mock infection. Following the same protocol as for measuring CD163 expression, cells were stained with monoclonal antibodies 18 hours post (mock) infection to assess their expression of CD169 (clone 41D3, University of Ghent) using flow-cytometry. However, in contrast to CD163, double staining of cells with monoclonal antibodies for both PRRSV infection and receptor expression was not possible in the case of CD169.

These additional replicates provided thus for every individual pig of batches 2 and 3 in addition to measures of PAM susceptibility and CD163 expression, longitudinal measures of the proportions of CD169 positive cells from the mock and PRRSV infected samples, respectively,

at the seven sampling times. Trends in CD169 expressions were analysed using linear mixed models using SAS *proc mixed* (SAS 9.3) with the same fixed and random effects as outlined in the main article.

**Results and Conclusions:**

Similar to CD163, the percentage of PAMs expressing CD169 on the cell surface showed an overall declining trend with increasing incubation time (Fig. S1A & B). The overall LSM percentage of CD169 positive cells declined from 75% (SE = 0.03) at day 0 to 34% (SE = 3) at day 9 with trend differences between infection groups (PRRSV and mock infection) and batches ($p<0.05$). The observed discrepancies between the time trends in susceptibility (Figure S1C) and in the expression of CD169 (Fig. S1A) and CD163 (Fig. S1B) indicate that changes in host cell susceptibility to PRRSV cannot be fully regulated by CD163 and CD169. In particular, at incubation day 0, the vast majority of PAMS expressed both molecules (Figs. S1A&B), but less than 10% of cells had become infected (Fig. S1C). In contrast, on day 6 of batch 3, the majority of cells expressed neither receptor at detectable levels yet 80% of cells had become infected.

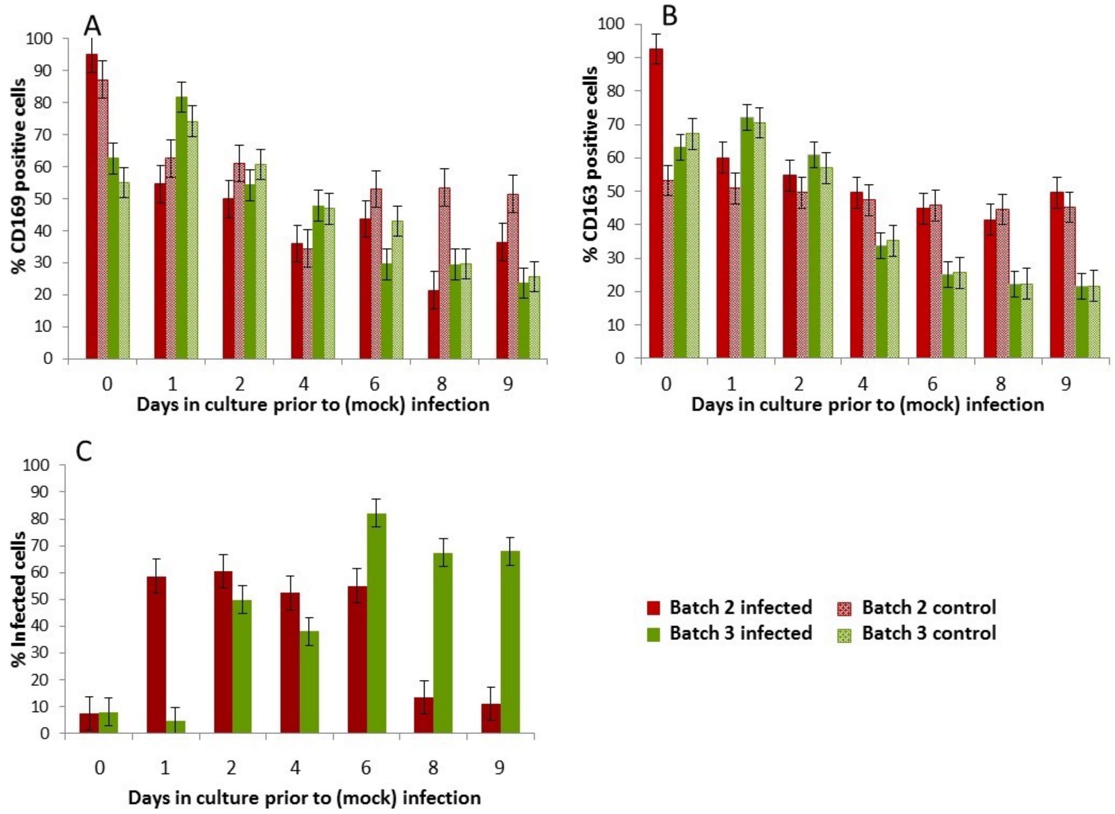


**Fig S1. CD169, CD163 biomarker and susceptibility dynamics observed in batches 2 and 3 of the in-vitro experiment.** (A) Percentage of PAMs classified as CD169 positive, (B) Percentage of CD163 positive PAMs, (C) Percentage of PAMs classified as susceptible to PRRSV, after being placed in culture for 0,1,2,4,6,8 and 9 days before infecting them with PRRSV (infected) or with mock-infection (panels A and B only). Measurements were taken 18 hours post (mock) infection. The control group denotes the mock-infected samples. The bars show the corresponding Least Square Mean (LSM) percentages of PAMs for each individual batch together with the standard errors. Results for the percentage of CD163 positive and infected cells are identical to those shown in the main article.
