# Supplementary figures and images for: Combining laboratory and mathematical models to infer mechanisms underlying kinetic changes in macrophage susceptibility to an RNA virus
Source: BMC Syst Biol. 2016 Oct 22;10:101. doi: 10.1186/s12918-016-0345-5 (PMC5075420; doi:10.1186/s12918-016-0345-5)

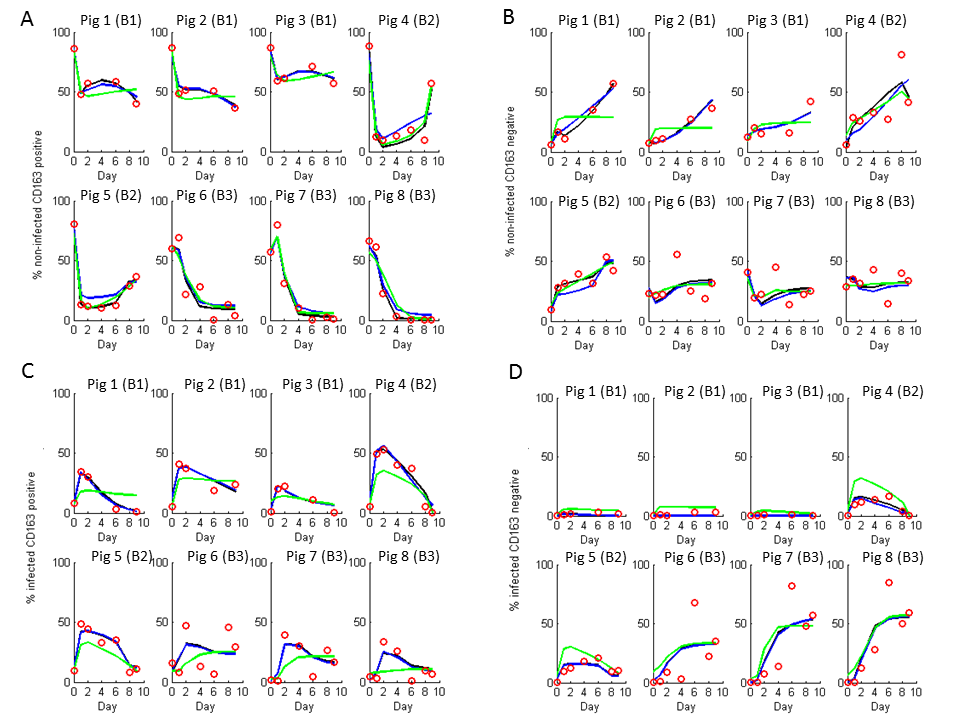

Supplement: Additional file 7: — Comparison of fits of various refined versions of mathematical model B to the experimental data for eight pigs from 3 batches (B1–B3). The various versions of model B refer to different assumptions regarding the role of CD163 for PAM susceptibility. Black lines refer to the assumption AS1 in the main article (i.e. CD163 does not affect the rates of switching between M− and M+, but directly enhances susceptibility of M+ cells to PRRSV (i.e. β1 < β2)). Blue lines represent the assumption AS2 (i.e. CD163 has neither direct nor indirect influence on PAM susceptibility). Green lines refer to assumption AS3 (i.e. the susceptibility state M has no influence on the differentiation rate from CD163 negative to CD163 positive state). Results for assumption AS4 in the main text are not plotted as they resulted in poor model fit and obstructed visibility of other model fits. Experimental data are represented by red circles. (A&B): percentage of non-infected PAMs classified as CD163 positive (A) and CD163 negative (B), respectively; (C&D): percentage of infected PAMs classified as CD163 positive (C) and CD163 negative (D), respectively. The fit statistics supported the assumption AS1 over AS2 (i.e. black lines fit better than blue lines; CD163 has no effect on the rate of switching between the susceptible and non-susceptible M states, but affects PAM susceptibility) for pigs 1, 4 and 5, but favoured AS2 over AS1 (blue lines over black lines) for the remaining pigs. Models incorporating AS3 (green lines) resulted in a significantly poorer model fit for all pigs than models representing AS1 and AS2 (black and blue lines). Models incorporating AS4 led to a significantly poorer model fit than those incorporating assumptions AS1─AS3 for all pigs. (TIF 273 kb) [file 12918_2016_345_MOESM7_ESM.tif]
