# Supplementary material for: Combining laboratory and mathematical models to infer mechanisms underlying kinetic changes in macrophage susceptibility to an RNA virus
Source: BMC Syst Biol. 2016 Oct 22;10:101. doi: 10.1186/s12918-016-0345-5 (PMC5075420; doi:10.1186/s12918-016-0345-5)
Supplement: Additional file 8: — Additional tables to assess the predictive value of models A and B. Tables showing the predictive value of models A and B based on RMSE and total bias. (PDF 389 kb) [file 12918_2016_345_MOESM8_ESM.pdf]

Additional File 7: Additional tables to assess the predictive value of models A and B

Table A: Predictive ability of models A and B, assessed by the $RMSE$ statistics, averaged over all pairs of individuals of the predictor and validation batches, respectively. Small RMSE refers to high predictive value.

	Model A			Model B		
Validation set / Predictor set	Batch 1	Batch 2	Batch 3	Batch 1	Batch 2	Batch 3
Batch 1	0.12	0.17	0.32	0.10	0.15	0.32
Batch 2	0.25	0.15	0.28	0.27	0.09	0.28
Batch 3	0.25	0.24	0.20	0.25	0.24	0.12

Table B: Predictive ability of models A and B, assessed by the total bias, averaged over all pairs of individuals of the predictor and validation batches, respectively.

	Model A			Model B		
Validation set / Predictor set	Batch 1	Batch 2	Batch 3	Batch 1	Batch 2	Batch 3
Batch 1	0.016	1.79	2.77	0.020	1.79	2.77
Batch 2	0.50	0.28	2.00	0.36	-0.02	1.80
Batch 3	-0.54	0.60	0.55	-0.83	0.14	-0.023

For the definition of RMSE and total bias, see main manuscript.
